# Regulatory Mechanism of CsMYB1‐CsMYB82/CsbHLH48‑*CsCAD4* Model for Resistance Against *Colletotrichum gloeosporioides* in *Camellia sinensis*

**DOI:** 10.1111/pbi.70659

**Published:** 2026-04-03

**Authors:** Rui Han, Yu Wang, Zekun Xue, Wang Wei, Cunqiang Ma, Jiake Zheng, Wuchen Yin, Zhen Zhao, Shujing Liu, Yuhua Wang, Jing Zhuang, Xuan Chen, Shifu Ma, Qiwei Huang, Xinghui Li

**Affiliations:** ^1^ College of Horticulture Nanjing Agricultural University Nanjing China; ^2^ State Key Laboratory for Crop Stress Resistance and High‐Efficiency Production/Key Laboratory of Horticultural Plant Biology and Germplasm Innovation in Northwest China, Ministry of Agriculture/College of Horticulture Northwest A&F University Yangling China; ^3^ Tea Industry Development Service Center of Wenxian Longnan China; ^4^ College of Resources and Environmental Sciences Nanjing Agricultural University Nanjing China

**Keywords:** *Camellia sinensis*, *Colletotrichum gloeosporioides*, CsMYB82, lignin

## Abstract

Anthracnose caused by *Colletotrichum gloeosporioides* is a major threat to tea cultivation; however, the molecular mechanism underlying different resistance among tea cultivars remains unclear. We identified distinct expression patterns of *CsMYB82* between anthracnose‐resistant and susceptible varieties after infection with anthracnose from previous RNA‐seq data. We further investigated the role of *CsMYB82* within a lignin‐associated regulatory network during anthracnose responses. We found that *CsMYB1* negatively regulates *CsMYB82* expression by Y1H screen. Additionally, we identified the interaction between CsMYB82 and CsbHLH48 both in vitro and in vivo. DNA‐affinity purification sequencing (DAP‐seq) revealed that *CsMYB82* directly binds to the promoter of *CsCAD4*, and this binding activity is enhanced in the presence of CsbHLH48. Functional analyses indicated that overexpression of *CsMYB82* or *CsCAD4* in tobacco and tea leaves was associated with increased susceptibility to anthracnose, whereas transient silencing of *CsMYB82* or *CsCAD4* via virus‐induced gene silencing (VIGS) in tea leaves resulted in reduced disease symptoms accompanied by elevated lignin accumulation. The functional analysis of CsMYB1 showed the opposite phenotype. Collectively, these results suggest that *CsMYB82* participates in a transcriptional regulatory module involving *CsMYB1*, CsbHLH48, and *CsCAD4*, which modulates lignin biosynthesis and influences anthracnose responses in tea plants. This study provides mechanistic insights into the transcriptional regulation of lignin‐associated defence responses and contributes to a better understanding of anthracnose resistance in tea.

## 1 Introduction

The susceptibility of tea plants ( *Camellia sinensis* (L.) O. Kuntze) to pests and pathogens is notably influenced by their need for specific levels of humidity and temperature, especially regarding anthracnose (Crous et al. 2004; Guo et al. 2014). The production of tea is adversely affected by *Colletotrichum*, which usually inhabits the buds, leaves, and branches of tea plants (Chen et al. 2017; Manawasinghe et al. 2021). The progression of anthracnose infection in tea leaves can be divided into three stages. In the early stage, cinnamon appears at the tip or edge of the leaves infected by the conidia of *Colletotrichum*. In the middle stage, the lesions formed irregular light brown and grey plaques. Finally, black flat round spots emerge on the lesions while leaf dehydration gradually progresses leading to brittleness and fragility. Ultimately, infected young and mature leaves both shed (Fang et al. 2013; Hu et al. 2021). Currently, chemical agents are the primary method to manage anthracnose in practical cultivation. However, this approach increases production costs, environmental pollution, and potential food safety risks (Jun et al. 2010; Chen et al. 2022). Identifying the genes and molecular pathways associated with resistance in tea plants would develop genetic resistance against anthracnose.

Numerous studies have highlighted the crucial role of lignin in plant disease resistance. As a physical barrier, lignin hinders the invasion of fungal pathogens. Strengthening the lignification in plant cell walls significantly enhances the plant's defence mechanisms against fungal pathogens (Park et al. 2019). Pathogen‐resistant tomato varieties exhibit a significantly higher lignin content compared to susceptible varieties (Mandal et al. 2013). In cotton, overexpression of GhDIR1, the gene involved in lignin deposition, promotes lignification in transgenic plants to enhance resistance against *Verticillium dahlia* (Shi et al. 2012). The transcriptome sequencing of tea plants has revealed that salicylic acid (SA) treatment can increase lignin accumulation in leaves, thus enhancing plant defence and improving disease resistance (Liu, Wang, Li, et al. 2023). Additionally, the application of 24‐epibrassinolide (EBR) can enhance the tea plants' resistance against *Colletotrichum fruticola* by promoting lignin synthesis (Zhang, Zhang, et al. 2023). However, the molecular mechanism through which lignin contributes to resistance to tea anthracnose remains unclear and requires further investigation.

Cinnamyl alcohol dehydrogenase (CAD), the key enzyme in lignin synthesis, catalyses the final step of the monolignol biosynthesis pathway by reducing hydroxycinnamaldehydes to their corresponding alcohols. CAD plays a crucial role not only in plant growth and development, but also in defence mechanisms against pathogens. The silencing of *CmCAD2* and *CmCAD3*, either individually or together, can significantly reduce CAD activity and lignin deposition, resulting in a dwarf phenotype (Liu et al. 2020). In *Cassava*, the physical interaction of MeRAV5 protein with *MeCAD15* promotes its activity to affect lignin accumulation (Yan et al. 2021). Furthermore, the heterologous expression of *PpCAD1* in *Arabidopsis* by increasing lignin content not only results in robust root systems, vigorous seedlings, and early flowering, but also enhances resistance against pathogens (Jiang et al. 2022). However, in Arabidopsis, only AtCAD4 and AtCAD5 were confirmed as having significant activity against cinnamyl aldehydes (Kim et al. 2004). AtCAD7 and AtCAD8 were shown to be strongly induced by pathogens and pathogen‐derived elicitors (Kiedrowski et al. 1992). And AtCAD8 exhibited a clear preference for 2‐methoxybenzaldehyde, showing substantially lower activity toward 3‐methoxybenzaldehyde, salicylaldehyde, benzaldehyde, and cinnamaldehyde (Somssich et al. 1996; Vega‐Arreguín et al. 2014).

The MYB transcription factors can significantly regulate phenylpropanoid biosynthesis, specifically the synthesis of key components of secondary cell walls such as lignin and cellulose (Stracke et al. 2001). The overexpression of *CmMYB19* enhances accumulation of lignin, thereby restricting the reproduction of *Macrosophoniella sanborni* on *Chrysanthemums* (Wang, Sheng, et al. 2017). *AtMYB15* activates lignin biosynthesis genes, such as PAL, C4H, 4CL, and CAD, resulting in reduced lignin deposition and compromised disease resistance in *myb15* mutant plants when infected with *Pst DC3000* in *Arabidopsis* (Kim et al. 2020). Although quantitative expression analysis and enzyme activity assays have demonstrated the involvement of MYB transcription factors in the regulation of lignin biosynthesis in tea plants (Li et al. 2017, 2022; Han, Yang, et al. 2021), further investigation into the specific mechanisms is still required.

Numerous ongoing studies have been conducted to investigate tea plant's resistance to *Colletotrichum*. Several transcription factors, belonging to the NAC, ERF, and MYB families, are induced upon *Colletotrichum* inoculation (Wang et al. 2016; Jeyaraj et al. 2019). In this study, we investigated the function of the R2R3‐MYB transcription factor CsMYB82 in tea plants and explored its regulatory role during anthracnose infection. Our results showed that overexpression of *CsMYB82* was associated with reduced lignin accumulation in both transgenic tobacco and tea leaves, accompanied by increased susceptibility to anthracnose. In contrast, transient suppression of CsMYB82 led to enhanced resistance, coinciding with elevated lignin levels. We further identified upstream regulators, interacting proteins, and downstream target genes of CsMYB82, revealing a transcriptional regulatory framework linking CsMYB82 to lignin‐associated responses during pathogen infection. Together, these findings suggest that CsMYB82 participates in the regulation of anthracnose responses in tea plants, likely through modulation of lignin biosynthesis, and provide useful insights for understanding the molecular basis of disease resistance in tea plants.

## 2 Results

### 2.1 Lignin Variation in Tea Cultivars With Different Resistance to Anthracnose

**FIGURE 1:**
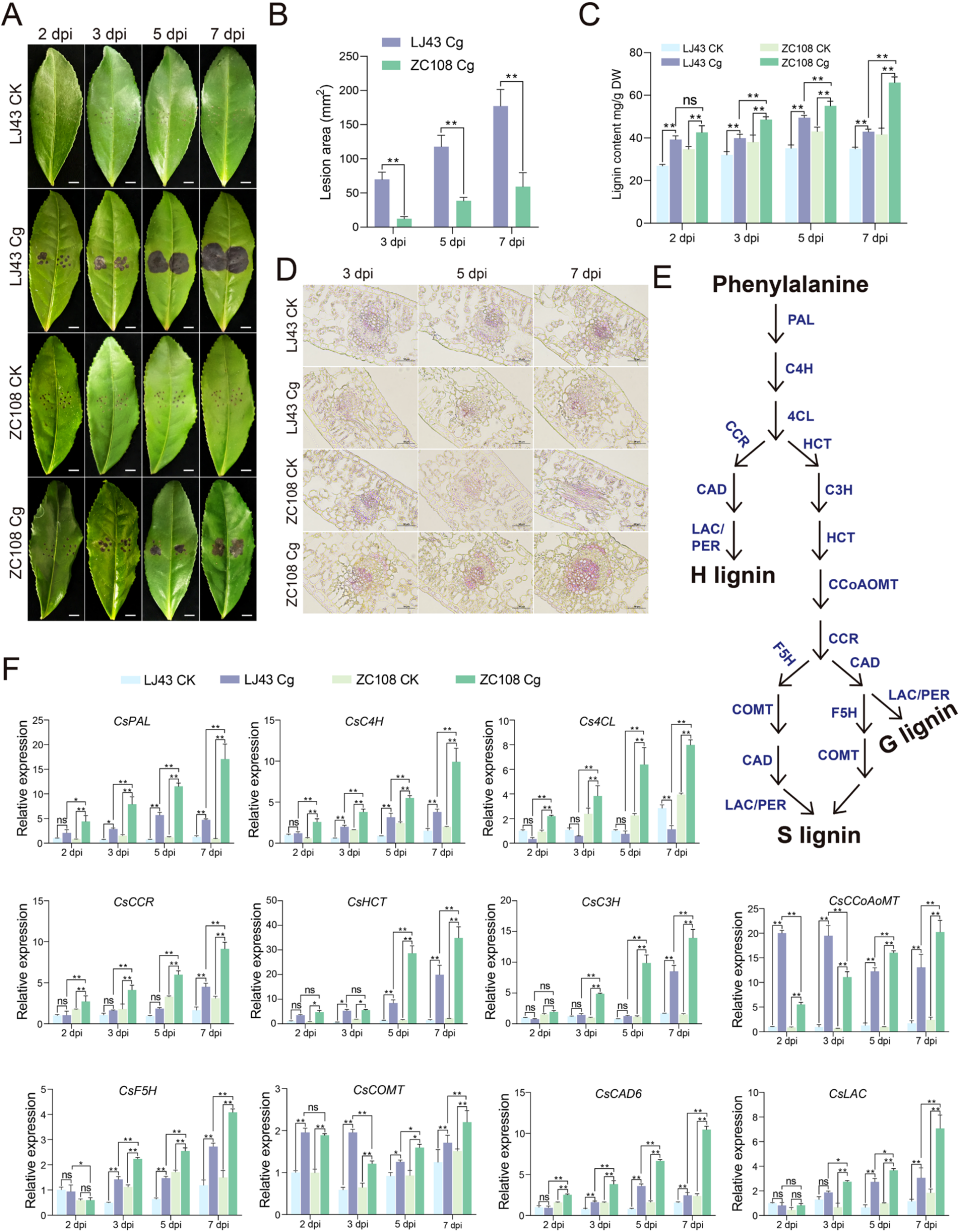
Disease symptom and determination of lignin in ‘Longjing 43’ and ‘Zhongcha 108’ after inoculation with *C. gloeosporioides*. (A) Disease symptom on leaves after *C. gloeosporioides* inoculation on two varieties of tea plant leaves (LJ43 and ZC108). LJCg, ZCCg (*C. gloeosporioides*–inoculated leaves) and CK (H_2_O‐inoculated control) after 2, 3, 5 and 7 days post inoculation (dpi). Scale bar = 2 cm. (B) The lesion areas of the leaves of ‘Longjing 43’ and ‘Zhongcha 108’ following inoculation with *C. gloeosporioides*. Values are presented as the means ± SD (*n* = 4). “ns” means no difference and asterisks indicate statistical significance using a Tukey's multiple comparison test followed by two‐way ANOVA (*p < 0.05, **p < 0.01). (C) Measurement of the total lignin content in leaves after infection with *C. gloeosporioides*. Values are presented as the means ± SD (*n* = 3). “ns” means no difference and asterisks indicate statistical significance using a Tukey's multiple comparison test followed by two‐way ANOVA (**p < 0.01). (D) Lignin accumulation through phloroglucinol staining at 3, 5 and 7 days post inoculation (dpi) on ‘Longjing 43’ and ‘Zhongcha 108’. (E) Schematic diagram of lignin metabolic pathway. (F) Expression analysis of lignin metabolism related genes by RT‐qPCR after *C. gloeosporioides* inoculation. The RT‐qPCR data were presented as means ± SD values with three biological replicates. “ns” means no difference and asterisks indicate statistical significance using a Tukey's multiple comparison test followed by two‐way ANOVA (*p < 0.05, **p < 0.01).

Pathogenicity assays showed that disease progression progressed over time in both cultivars after *C. gloeosporioides* inoculation, whereas the mock controls (CK) showed no obvious lesions. Compared with ‘ZC108’, ‘LJ43’ developed markedly more severe necrotic lesions, and lesion expansion was significantly faster in ‘LJ43’ (Figure 1A). Quantification confirmed that the lesion area in ‘LJ43’ was significantly larger than that in ‘ZC108’ at 3, 5, and 7 dpi (Figure 1B). In parallel, infection triggered lignin accumulation in both cultivars (Figure 1C). Although lignin contents increased upon inoculation, ‘ZC108’ exhibited a stronger lignification response, showing significantly higher lignin levels than ‘LJ43’ at later stages, particularly at 7 dpi. Histochemical staining of leaf cross‐sections further supported these results (Figure 1D), revealing enhanced lignin‐associated staining in vascular tissues after infection, which was more pronounced in ‘ZC108’ than in ‘LJ43’ as disease progressed. Quantitative analysis showed that most lignin biosynthesis‐related genes were significantly more highly expressed in ‘ZC108’ than in ‘LJ43’ during anthracnose infection, except *CsCCoAOMT* and *CsCOMT*. Notably, the expression levels of lignin pathway genes in ‘ZC108’ were consistently higher and maintained over a longer infection period, indicating a sustained activation of lignin biosynthesis. In contrast, ‘LJ43’ exhibited a delayed and transient transcriptional response, with lignin biosynthesis‐related genes showing lower induction levels and a shorter duration of expression compared with ‘ZC108’ (Figure 1F).

### 2.2 CsMYB82 Characterisation and Its Role in Promoting *C. gloeosporioides* Infection via Lignin Reduction in Transgenic Tobacco

**FIGURE 2:**
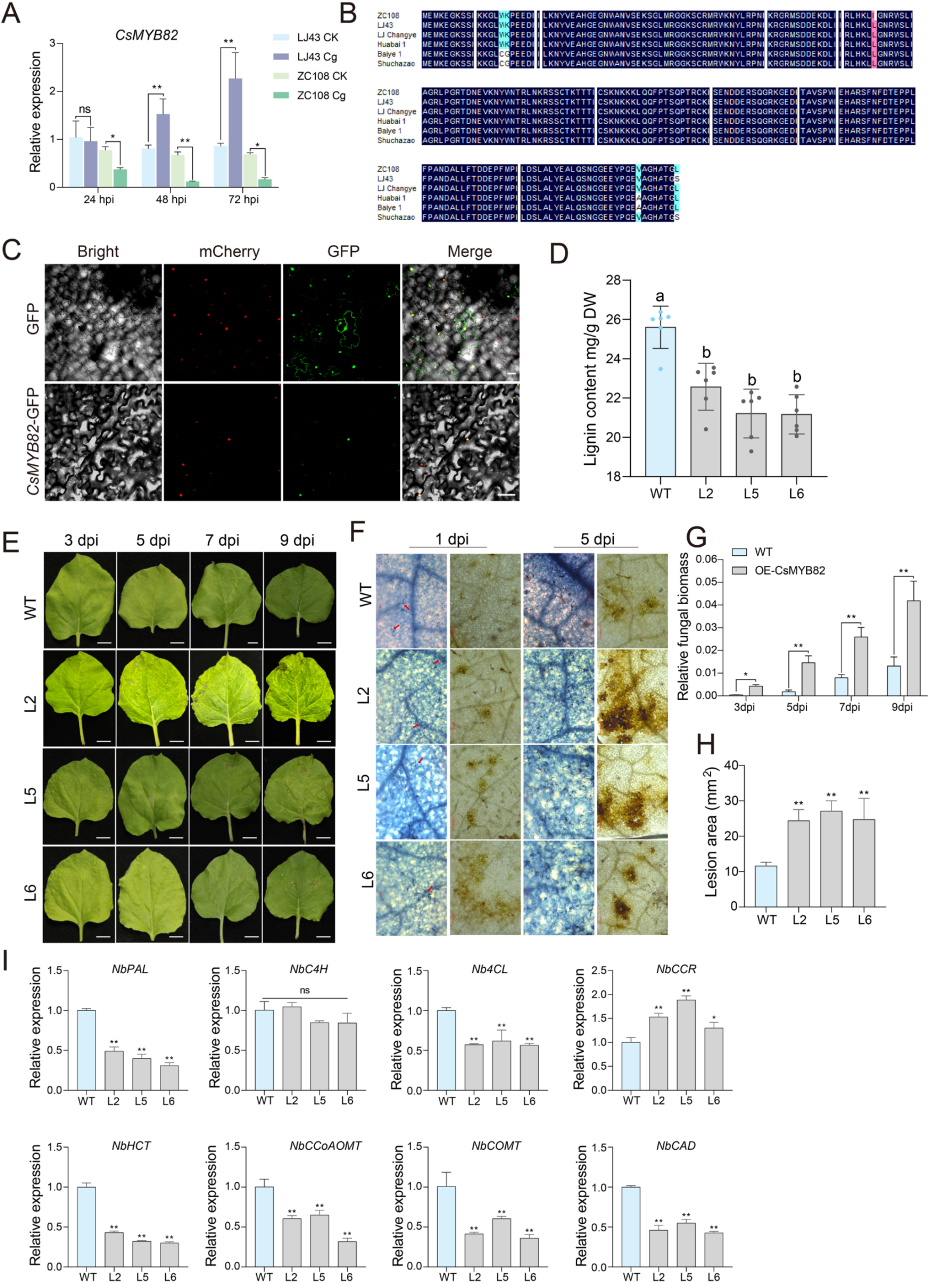
Bioinformatic analyses and overexpressing *CsMYB82* in tobacco enhance susceptibility to anthracnose. (A) Expression analysis of *CsMYB82* by RT‐qPCR at 24, 48 and 72 h post inoculation (hpi) after *C. gloeosporioides* inoculation. Three independent replicates were conducted for each RT‐qPCR analysis. Asterisks indicate statistical significance using a Tukey's multiple comparison test followed by two‐way ANOVA (*p < 0.05, **p < 0.01). (B) Different tea varieties protein sequence alignment of CsMYB82. Differences are highlighted in blue. (C) Subcellular localisation of CsMYB82 in tobacco leaves. Bar = 50 μm. (D) Measurement of the total lignin content in transgenic *CsMYB82* lines (L2, L5, L6) and wild type (WT) leaves. Values are presented as the means ± SD (*n* = 6). Different letters above the bars denote significant differences using a Tukey's multiple comparison test followed by one‐way ANOVA (*p* < 0.05). (E) Evaluation of anthracnose resistance levels in CsMYB82‐ overexpression lines (L2, L5, L6) and wild type (WT) tobacco at 3, 5, 7, 9 days post inoculation (dpi) with *C. gloeosporioides*. Scale bar = 1 cm. (F) The hyphae growth of *C. gloeosporioides* (Scale bar = 100 μm) and H_2_O_2_ accumulation (Scale bar = 200 μm) in leaves of transgenic *CsMYB82* lines (L2, L5, L6) and wild type (WT) at 1, 5 days post inoculations (dpi) by trypan blue and DAB staining. Red arrows indicate hyphae of *C. gloeosporioides*. (G) qPCR analysis of fungal biomass in different mixtures of OE‐*CsMYB82* lines (L2, L5, L6) and the WT plants at 3, 5, 7, 9 days post inoculation (dpi) with *C. gloeosporioides*. Values are presented as the means ± SD (*n* = 3). Asterisks indicate statistical significance using a Tukey's multiple comparison test followed by two‐way ANOVA (*p < 0.05, **p < 0.01). (H) The lesion areas of transgenic *CsMYB82* lines (L2, L5, L6) and wild type (WT) at 9 day post inoculation (dpi). Values are presented as the means ± SD (*n* = 3). Asterisks indicate statistical significance using Tukey's multiple comparison test followed by one‐way ANOVA (**p < 0.01). (I) Expression analysis of lignin metabolism related genes by RT‐qPCR after *C. gloeosporioides* inoculation. The RT‐qPCR data were presented as means ± SD values with three biological replicates. “ns” means no difference and asterisks indicate statistical significance using a Tukey's multiple comparison test followed by one‐way ANOVA (*p < 0.05, **p < 0.01).

The transcription factor *CsMYB82* was identified from transcriptome data (Jeyaraj et al. 2021) as differentially expressed after *C. gloeosporioides* infection. In the susceptible cultivar ‘LJ43’, CsMYB82 expression was significantly induced, whereas CsMYB82 expression was suppressed in the resistant cultivar ‘ZC108’ (Figure 2A). The ORF of *CsMYB82* was cloned from ‘ZC108’, with a full length of 687 bp encoding 229 amino acids. It is located on chromosome 6 and contains a SANT domain, classifying it as a typical R2R3‐MYB transcription factor (Figure S1A,B). Sequence comparison among different tea cultivars revealed only four amino acid differences (Figure 2B), and phylogenetic analysis indicated that *CsMYB82* from ‘ZC108’ is most closely related to *ClMYB82* from *Camellia lanceoleosa* (Figure S2A). No significant transcriptional self‐activation activity was observed (Figure S2B). Subcellular localisation showed nuclear localisation (Figure 2C).

Transgenic tobacco lines with constitutive overexpression of *CsMYB82* were generated to examine its role in disease resistance. RT‐qPCR confirmed elevated expression levels in homozygous lines (Figure S2C,D). Lignin content measurements showed that all three overexpression lines had significantly lower lignin levels compared to WT (Figure 2D). At 3 dpi after *C. gloeosporioides* infection, no visible symptoms were observed in either WT or transgenic leaves. From 5 to 9 dpi, transgenic leaves exhibited gradually enlarging lesions, whereas only small lesions appeared on WT leaves by 9 dpi (Figure 2E). Trypan blue staining revealed increasing fungal hyphal density in the transgenic lines over time. DAB staining further showed higher H_2_O_2_ accumulation in transgenic leaves compared to WT upon infection (Figure 2F). The fungal biomass of OE‐*CsMYB82* was significantly higher than that of the WT, and the lesion area was also significantly larger than that of the WT (Figure 2G,H). In OE‐*CsMYB82*, the lignin metabolism‐related genes showed no significant changes in *NbC4H*, while *NbCCR* significantly increased. Compared to the WT, the other six genes were all significantly downregulated (Figure 2I). These results indicate that *CsMYB82* overexpression in tobacco is associated with reduced lignin accumulation and increased susceptibility to *C. gloeosporioides* infection.

### 2.3 *CsMYB82* Enhanced the Sensitivity of Tea Plant Leaves to Anthracnose

**FIGURE 3:**
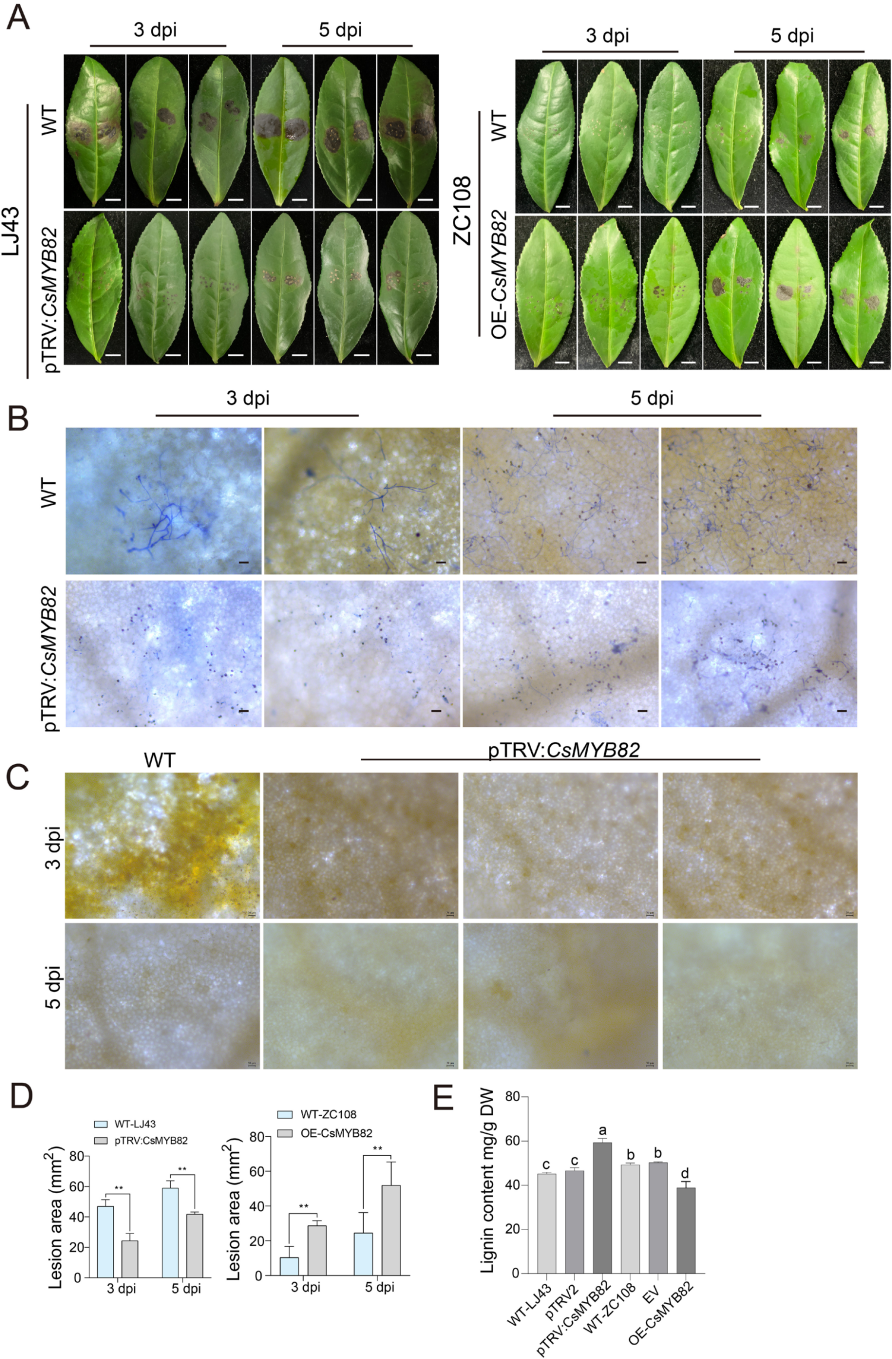
*CsMYB82* is a negative regulator of anthracnose resistance in *Camellia sinensis*. (A) Disease symptom on pTRV: *CsMYB82* and OE‐*CsMYB82* leaves after *C. gloeosporioides* inoculation at 3, 5 days post inoculation (dpi) in ‘Longjing 43’ and ‘Zhongcha 108’ leaves. Scale bar = 2 cm. (B) The hyphae growth of *C. gloeosporioides* in leaves of pTRV: *CsMYB82* leaves and wild type (WT) at 3, 5 days post inoculation (dpi) by trypan blue. Scale bar = 30 μm. (C) The H_2_O_2_ accumulation in leaves of pTRV: *CsMYB82* leaves and wild type (WT) at 3 and 5 days post inoculation (dpi) by DAB staining. Scale bar = 30 μm. (D) The lesion areas of pTRV: *CsMYB82*, OE‐*CsMYB82* and wild type (WT‐LJ43, WT‐ZC108) leaves at 3 and 5 days post inoculation (dpi). Values are presented as the means ± SD (*n* = 3). Asterisks indicate statistical significance using Tukey's multiple comparison test followed by two‐way ANOVA (**p < 0.01). (E) Measurement of the total lignin content in pTRV: *CsMYB82*, OE‐*CsMYB82* and wild type (WT‐LJ43, WT‐ZC108) leaves after infiltration at 72 h. Values are presented as the means ± SD (*n* ≥ 6). Different letters above the bars denote significant differences using a Tukey's multiple comparison test followed by one‐way ANOVA (*p* < 0.05).

To elucidate the role of *CsMYB82* in tea plant defence against anthracnose, we first employed virus‐induced gene silencing (VIGS) to transiently suppress *CsMYB82* expression. Transcript levels of *CsMYB82* were significantly reduced in pTRV: *CsMYB82* leaves, as confirmed by RT‐qPCR (Figure S2E,F). Upon inoculation with *C. gloeosporioides*, pTRV: *CsMYB82* plants remained symptom‐free at 3 dpi, while WT leaves displayed clear necrotic lesions. By 5 dpi, disease progression remained limited in pTRV: *CsMYB82* plants, but continued to advance in WT (Figure 3A). The measurement of lesion area also supports the phenotypic characteristics (Figure 3D). Trypan blue staining revealed abundant mature hyphae in WT leaves, whereas fungal structures in pTRV: *CsMYB82* leaves were largely restricted to haustoria and short hyphae (Figure 3B). DAB staining revealed that at 3 dpi, WT leaves showed pronounced yellow deposition, whereas pTRV: *CsMYB82* leaves displayed markedly weaker staining. By 5 dpi, the deposition was largely diminished in both WT and pTRV: *CsMYB82* leaves, with only sporadic residual patches observed in pTRV: *CsMYB82* leaves (Figure 3C). Notably, lignin content was significantly higher in silenced leaves than in WT (Figure 3E), consistent with upregulated expression of several lignin biosynthesis genes, except for *Cs4CL* (Figure S3A).

To complement the silencing assay, we transiently overexpressed *CsMYB82* in tea leaves via *Agrobacterium*‐mediated infiltration. RT‐qPCR confirmed elevated *CsMYB82* transcript levels in OE‐*CsMYB82* leaves (Figure S2G). In contrast to silenced leaves, OE‐*CsMYB82* plants showed earlier lesion development and a greater extent of disease symptoms following *C. gloeosporioides* infection (Figure 3A,D). Phloroglucinol‐HCL staining and lignin quantification both demonstrated reduced lignin accumulation in OE‐*CsMYB82* leaves relative to WT and EV controls (Figures S2H and 3E). Apart from *Cs4CL*, all genes involved in the lignin biosynthetic pathway were upregulated in the ‐OE‐*CsMYB82* leaves (Figure S3B). Collectively, our findings support a role for CsMYB82 as a negative modulator of lignin accumulation, which correlates with changes in disease resistance during *C. gloeosporioides* infection.

### 2.4 *CsMYB1* Represses *CsMYB82* Expression That Enhances Tea Plant Resistance to Anthracnose

To investigate the transcriptional regulation of *CsMYB82*, we cloned a 1712 bp promoter region (*ProCsMYB82*) using genomic DNA from the tea cultivar ‘ZC108’ (Figure S4A). In silico cis‐element prediction via PlantCARE identified multiple core promoter elements, including CAAT boxes and AT–TATA boxes, as well as light‐responsive motifs (Box 4, Box II, GT1 motifs, chs‐CMA1a), hormone‐responsive elements (TCA element for salicylic acid and AAGAA motif for abscisic acid), and various stress‐related elements (STRE, WRE3, ARE). Putative MYB and MYC binding sites were also detected, alongside a single F‐box motif possibly associated with ubiquitin‐mediated regulation (Figure S4B).

**FIGURE 4:**
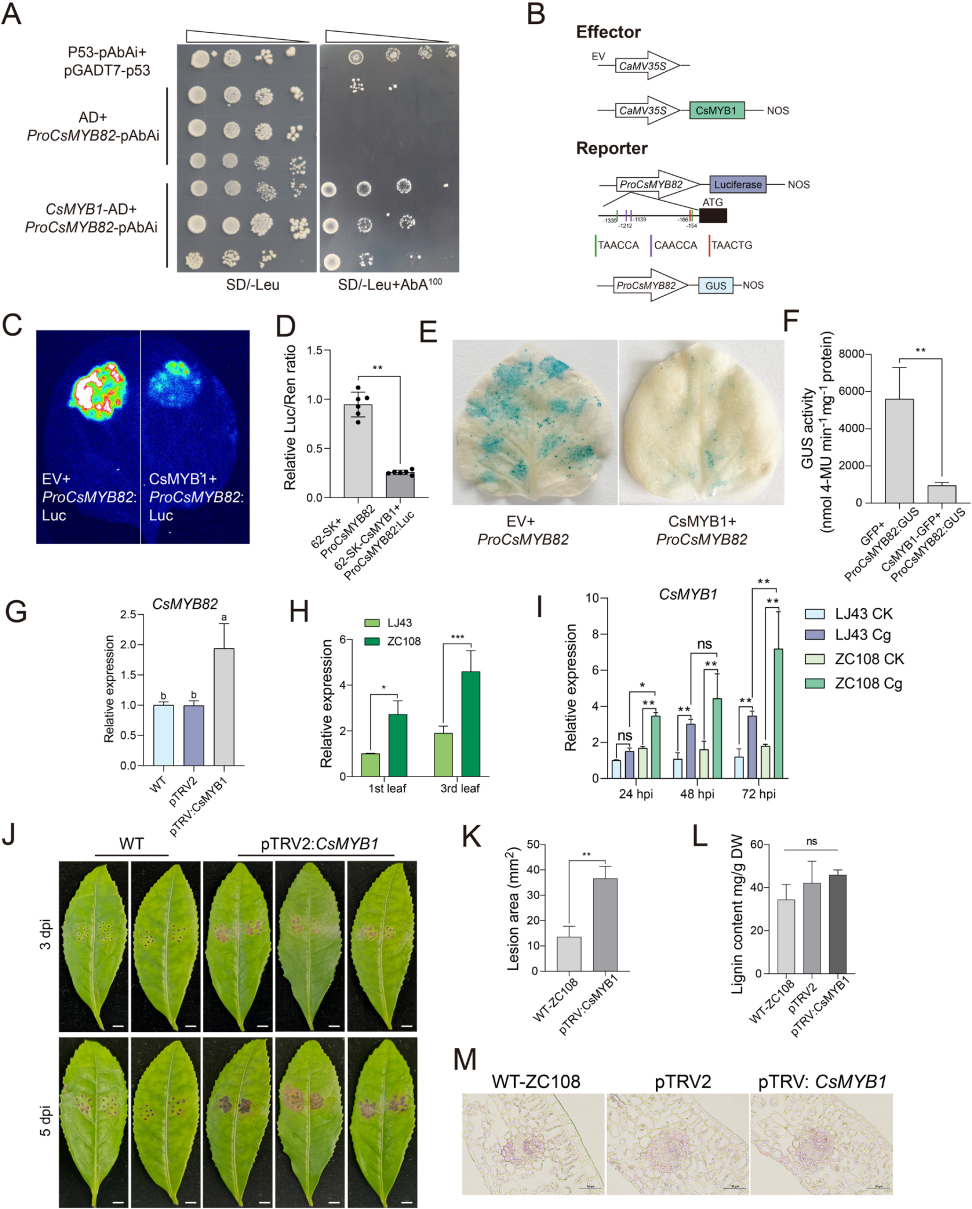
Identification and functional analysis of upstream regulatory genes of *CsMYB82*. (A) Yeast One‐Hybrid assay indicated CsMYB1 transcription factor specifically binds to *ProCsMYB82*. Yeast cultures were inoculated onto SD/−Leu media containing 100 ng mL^−1^ aureobasidin A. Y1HGold[p53‐AbAi] co‐transformed with pGADT7‐p53 was used as positive control. Y1HGold[pAbAi‐*ProCsMYB82*] co‐transformed with pGADT7 recombinant plasmid was used as negative control and test group. (B) Schematic diagram of *CsMYB82* promoters and the effector and reporter constructs. TAACCA, CAACCA and TAACTG are MYB‐binding cis‐elements. (C) Luciferase assay shows that CsMYB1 inhibits the expression of *CsMYB82* in *Nicotiana benthamiana* leaves. The *ProCsMYB82*‐Luc with pGreenII 62‐SK‐CsMYB1 and pGreenII 62‐SK empty vector was co‐transformed for the test and control, respectively. (D) Luciferase (Luc) assay indicated that CsMYB1 decreases *CsMYB82* promoter activity. The empty vector pGreenII 62‐SK and the *CsMYB82* promoter were set as 1. Values are presented as the means ± SD (*n* = 6). Asterisks indicate statistical significance using Tukey's multiple comparison test followed by one‐way ANOVA (**p < 0.01). (E) CsMYB1 negatively regulated the promoter activity of CsMYB82 by GUS Staining. The *ProCsMYB82*‐GUS with pCAMBIA2300‐CsMYB1‐GFP and pCAMBIA2300‐GFP empty vector was co‐transformed for the test and control, respectively. (F) Repression of transactivation of the CsMYB82 promoter by CsMYB1 for β‐glucuronidase (GUS) activity analysis. Asterisks indicate significant differences using the Student's *t*‐test (**p < 0.01). (G) Expression analysis of *CsMYB82* by RT‐qPCR on pTRV: *CsMYB1* leaves. The RT‐qPCR data were presented as means ± SD values with three biological replicates. Different letters above the bars denote significant differences using a Tukey's multiple comparison test followed by one‐way ANOVA (*p* < 0.01). (H) Expression analysis of *CsMYB1* by RT‐qPCR in the first and third leaf positions of ‘Longjing 43’ and ‘Zhongcha 108’ leaves. The RT‐qPCR data were presented as means ± SD values with three biological replicates and asterisks indicate statistical significance (*p < 0.05, **p < 0.01). (I) Expression analysis of *CsMYB1* by RT‐qPCR at 24, 48 and 72 h post inoculation (hpi) after *C. gloeosporioides* inoculation. Three independent replicates were conducted for each RT‐qPCR analysis. “ns” means no difference and asterisks indicate statistical significance using a Tukey's multiple comparison test followed by two‐way ANOVA (**p < 0.01). (J) Disease symptoms on pTRV: *CsMYB1* leaves after *C. gloeosporioides* inoculation at 3, 5 days post inoculation (dpi) in ‘Zhongcha 108’ leaves. Scale bar = 2 cm. (K) The lesion areas of pTRV: *CsMYB1* and wild type (WT‐ZC108) leaves at 5 day post inoculation (dpi). Values are presented as the means ± SD (*n* = 3). Asterisks indicate statistical significance using Tukey's multiple comparison test followed by one‐way ANOVA (**p < 0.01). (L) Measurement of the total lignin content in pTRV: *CsMYB1* and wild type (WT‐ZC108) leaves after infiltration at 72 h. Values are presented as the means ± SD (*n* = 3). “ns” means no difference using a Tukey's multiple comparison test followed by one‐way ANOVA. (M) Lignin accumulation of pTRV: *CsMYB1*, wild type (WT‐ZC108) and pTRV2 leaves through phloroglucinol‐HCL staining on ‘Zhongcha 108’.

To identify transcription factors that regulate *CsMYB82*, a yeast one‐hybrid (Y1H) screen was performed using the *ProCsMYB82* sequence fused into the pAbAi vector. The minimal inhibitory concentration of Aureobasidin A (AbA) was determined for screening (Figure S4C). Sequencing of positive clones revealed a candidate transcription factor with high sequence similarity to CsMYB1 (Table S3). Notably, *CsMYB1* is homologous to *AtMYB6* in *Arabidopsis thaliana* , which is implicated in responses to *Pseudomonas syringae* (Kranz et al. 1998), suggesting a potential role in disease resistance. To evaluate the direct binding of CsMYB1 to the *CsMYB82* promoter, a Y1H assay was conducted. Yeast cells co‐transformed with CsMYB1‐AD and *ProCsMYB82*‐pAbAi grew strongly on SD/−Leu medium containing 100 ng mL^−1^ AbA, whereas negative controls failed to grow, confirming the specific interaction between CsMYB1 and the *CsMYB82* promoter (Figure 4A). The *ProCsMYB82* harbours canonical MYB binding motifs (TAACCA, CAACCA, and TAACTG). To further validate this interaction *in planta*, a dual‐luciferase and GUS reporter system was used (Figure 4B). Co‐expression of 35S:*CsMYB1* with *ProCsMYB82*: Luc or *ProCsMYB82*: GUS in *Nicotiana benthamiana* epidermal cells revealed marked suppression of reporter activity by CsMYB1 (Figure 4C,E). LUC activity decreased by ~3.7‐fold in the presence of overexpressed *CsMYB1* (Figure 4D). The GUS staining and activity further supported this repression (Figure 4F). And the *CsMYB82* expression was enhanced in pTRV: *CsMYB1* leaves (Figure 4G).

Subcellular localisation revealed that *CsMYB1* was predominantly nuclear (Figure S5A). RT‐qPCR analysis showed under natural (non‐infected) conditions, *CsMYB1* transcript levels were consistently higher in ‘ZC108’ than in ‘LJ43’ in both the first and third leaf positions (Figure 4H). In addition, *CsMYB1* expression was rapidly induced in ‘ZC108’ as early as 24 hpi following *C. gloeosporioides* infection, whereas no significant change was detected in ‘LJ43’. At 48–72 hpi, *CsMYB1* expression in ‘ZC108’ was significantly higher than that in ‘LJ43’ (Figure 4I). Functional assays were performed by transient overexpression and silencing of *CsMYB1* in tea plant leaves (Figure S5B). RT‐qPCR confirmed effective alteration of *CsMYB1* expression (Figure S5C,D). Following *C. gloeosporioides* inoculation, lesion size and area in OE‐*CsMYB1* leaves was significantly reduced compared to WT at both 3 and 5 dpi (Figure S5E,F). And in OE‐*CsMYB1* leaves, the lignin content also increased (Figure S5G). Notably, *CsMYB82* expression was repressed in OE‐*CsMYB1* leaves (Figure S3H). Conversely, silencing of *CsMYB1* via VIGS in ‘ZC108’ resulted in enhanced disease severity (Figure 4J,K). Interestingly, the content of lignin did not change in pTRV: *CsMYB1* (Figure 4L). Consistently, phloroglucinol‐HCl staining showed no obvious difference in lignin accumulation between pTRV: *CsMYB1* leaves and controls, while OE‐*CsMYB1* leaves displayed stronger coloration, suggesting increased lignin deposition (Figures 4M and S5I). Collectively, these findings suggest that CsMYB1 acts as an upstream repressor of CsMYB82 and may contribute to enhanced anthracnose resistance in tea leaves by modulating transcriptional responses associated with stress and defence.

### 2.5 CsMYB82 Interacts With CsbHLH48

To explore potential interaction partners of *CsMYB82* in tea plant defence against anthracnose, a yeast two‐hybrid (Y2H) screen was performed. Although *CsMYB82* lacks transcriptional activation activity, 100 ng mL^−1^ AbA was used to increase selection stringency during library screening. Using CsMYB82‐BD as bait, 31 interacting clones were identified from a tea cDNA library (Table S4). Given the well‐established role of MYB‐bHLH transcriptional complexes in biotic stress responses (Tai et al. 2013; Ng et al. 2018), *CsbHLH48* was selected for further characterisation.

**FIGURE 5:**
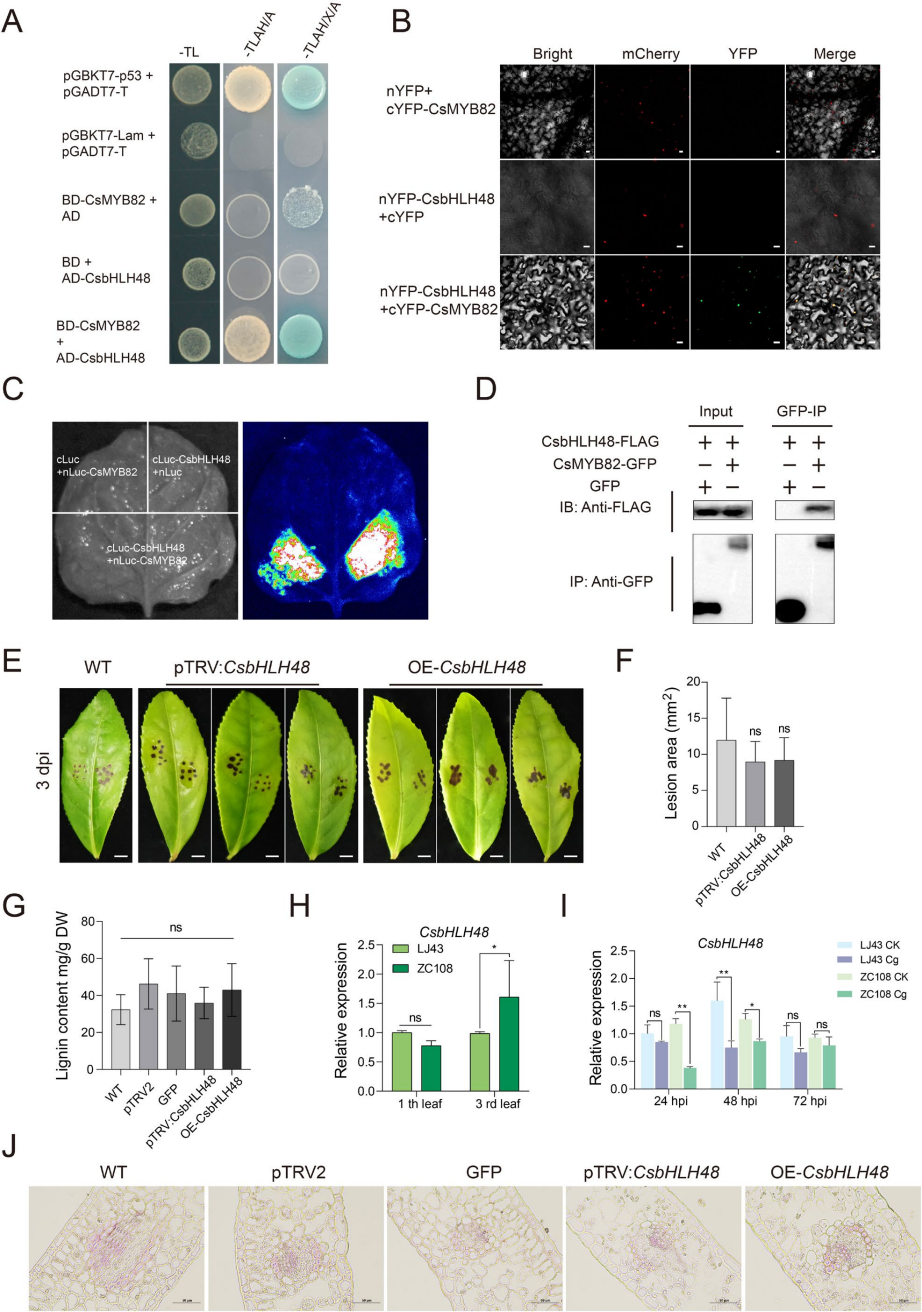
CsMYB82 interacts with CsbHLH48. (A) Yeast two‐hybrid assay. pGBKT7‐ CsMYB82 and pGADT7‐ CsbHLH48 plasmids were co‐transformed into Y2H Gold cells. pGBKT7‐p53 + pGADT7‐T, pGBKT7‐Lam + pGADT7‐T and pGBKT7‐ CsMYB82 + pGADT7 served as positive and negative controls, respectively. SD/−Trp‐Leu; SD/−Trp‐Leu‐His‐Ade/AbA; SD/−Trp‐Leu‐His‐Ade/X‐α‐Gal/AbA. (B) Bimolecular fluorescence complementation (BiFC) assay showing that CsMYB82 and CsbHLH48 interact in the nucleus of tobacco. Nuclei were counterstained with Marker mCherry. Scale bar = 20 μm. (C) Co‐immunoprecipitation assays (Co‐IP) showing the interaction between CsMYB82 and CsbHLH48. (D) Split‐luciferase (Luc) complementation assay of CsMYB82 and CsbHLH48 interaction in tobacco leaves. The controls were: NLuc + cLuc; nLuc‐ CsMYB82 + cLuc; nLuc + cLuc‐ CsbHLH48. Luminescence intensity showed the interaction of CsMYB82 and CsbHLH48 in tobacco leaves. (E) Disease symptom of pTRV: *CsbHLH48* and OE‐*CsbHLH48* leaves after *C. gloeosporioides* inoculation at 3 day post inoculation (dpi) on ‘Zhongcha 108’. Scale bar = 2 cm. (F) The lesion areas of pTRV: *CsbHLH48* and OE‐*CsbHLH48* leaves at 3 days post inoculations (dpi). Values are presented as the means ± SD (*n* = 3). “ns” means no difference using a Tukey's multiple comparison test followed by one‐way ANOVA. (G) Measurement of the total lignin content in pTRV: *CsbHLH48* and OE‐*CsbHLH48* leaves under natural conditions. Values are presented as the means ± SD (*n* ≥ 6). “ns” means no difference using a Tukey's multiple comparison test followed by one‐way ANOVA. (H) Expression analysis of *CsbHLH48* by RT‐qPCR in the first and third leaf positions of ‘Longjing 43’ and ‘Zhongcha 108’ leaves. The RT‐qPCR data were presented as means ± SD values with three biological replicates. “ns” means no difference and asterisks indicate statistical significance using a Tukey's multiple comparison test followed by two‐way ANOVA (*p < 0.05). (I) Expression analysis of *CsbHLH48* by RT‐qPCR at 24, 48 and 72 h post inoculation (hpi) after *C. gloeosporioides* inoculation. Three independent replicates were conducted for each RT‐qPCR analysis. “ns” means no difference and asterisks indicate statistical significance using a Tukey's multiple comparison test followed by two‐way ANOVA (*p < 0.05, **p < 0.01). (J) Lignin accumulation through phloroglucinol staining in pTRV: *CsbHLH48* and OE‐*CsbHLH48* leaves.

Yeast cells co‐transformed with pGBKT7‐CsMYB82 and pGADT7‐CsbHLH48 exhibited robust growth on SD/−Ade‐His‐Leu‐Trp medium and developed blue coloration in the presence of X‐α‐Gal and AbA, comparable to the positive control (Figure 5A), indicating a specific interaction between CsMYB82 and CsbHLH48 in yeast. This interaction was further validated in *N. benthamiana* leaves using bimolecular fluorescence complementation (BiFC). Co‐expression of CsMYB82‐nYFP and CsbHLH48‐cYFP yielded strong nuclear‐localised YFP fluorescence, confirming their interaction (Figure 5B). Co‐immunoprecipitation (CoIP) assays further substantiated this interaction: CsMYB82‐GFP efficiently pulled down CsbHLH48‐FLAG, while no interaction was observed with the GFP control (Figure 5D). Consistently, split luciferase complementation assays demonstrated strong luminescence signals in leaves co‐expressing nLUC‐CsMYB82 and cLUC‐CsbHLH48, but not in control treatments (Figure 5C).

To further explore the potential relevance of this interaction under infection conditions, we next analysed the expression patterns and functional effects of CsbHLH48 in tea plants. Subcellular localisation experiments revealed that CsbHLH48 is predominantly localised in the nucleus (Figure S6A). To investigate the role of CsbHLH48 in anthracnose resistance, transient overexpression and virus‐induced gene silencing (VIGS) assays were performed in tea leaves (Figure S6B). Following inoculation with *C. gloeosporioides*, neither OE‐*CsbHLH48* nor pTRV: *CsbHLH48* resulted in a significant change in disease severity compared with the controls at 3 dpi (Figure 5E,F). Consistently, lignin content analysis revealed no significant differences between OE‐CsbHLH48, pTRV: *CsbHLH48*, and control leaves, indicating that manipulation of CsbHLH48 alone does not markedly affect lignin accumulation during anthracnose infection (Figure 5G,J). And CsbHLH48 expression did not differ significantly between ‘LJ43’ and ‘ZC108’ in the first leaf position, whereas in the third leaf position, its expression level was significantly higher in the resistant cultivar ‘ZC108’ than in the susceptible cultivar under natural conditions ‘LJ43’ (Figure 5H). RT‐qPCR analysis showed that *CsbHLH48* expression was significantly suppressed in ‘ZC108’ at 24 hpi, whereas no significant change was observed in ‘LJ43’ at this early stage of infection. At 48 hpi, *CsbHLH48* expression was significantly repressed in both ‘ZC108’ and ‘LJ43’, indicating a general downregulation of CsbHLH48 during the progression of anthracnose infection. At 72 hpi, no significant difference was observed between ‘ZC108’ and ‘LJ43’ (Figure 5I).

### 2.6 CsMYB82 Direct DNA Targets Identified by DAP‐Seq

**FIGURE 6:**
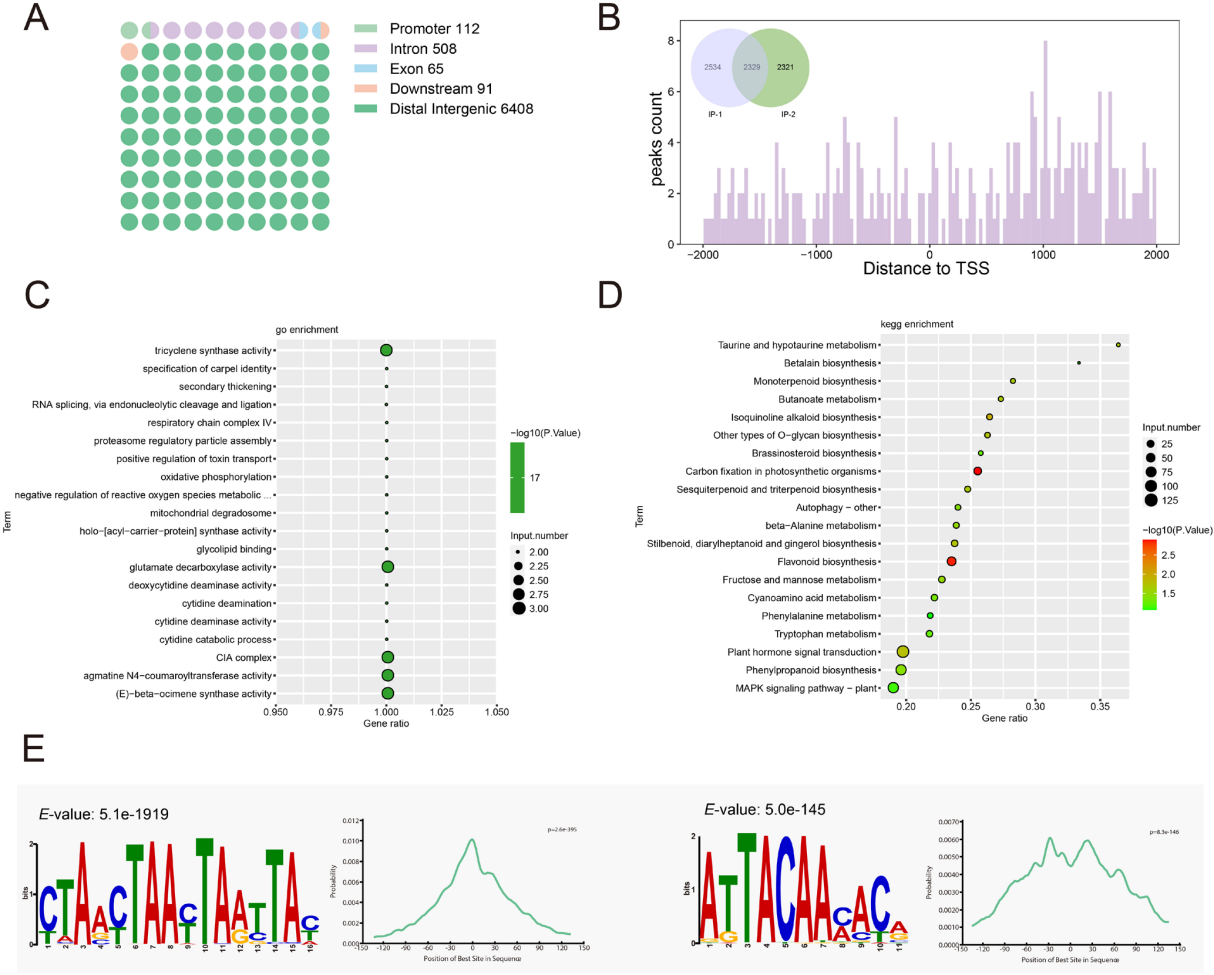
DAP‐seq detection of CsMYB82 binding target genes and sites. (A) Distribution of CsMYB82 binding sites. (B) Distribution of peaks at transcription start site (TSS) and analyse the consistency between the two techniques repeated Peak. (C, D) GO (C) and KEGG (D) analysis of CsMYB82 binding target genes. (E) Significantly enriched motif sequence of CsMYB82 binding sites was determined using the MEME suite. The *E*‐value indicates the statistical significance of the corresponding motif.

To identify genome‐wide DNA binding sites and direct target genes of *CsMYB82*, DNA affinity purification sequencing (DAP‐seq) was performed. Clean reads were aligned to the tea plant reference genome, and alignment statistics are provided in Table S5. A total of 7184 high‐confidence peaks were identified, with the majority (89.19%; 6408 peaks) located in distal intergenic regions. A smaller proportion of peaks were found within promoter regions (1.56%; 112 peaks) (Table S6), introns (7.07%; 508 peaks), exons (0.90%; 65 peaks), and downstream regions (1.27%; 91 peaks) (Figure 6A).

Among the identified peaks, 2329 were reproducibly detected in both biological replicates. These overlapping peaks were significantly enriched within 1000 bp of transcription start sites (TSS), indicating preferential binding of CsMYB82 near regulatory regions (Figure 6B). Gene Ontology (GO) enrichment analysis revealed that CsMYB82 target genes were associated with diverse molecular functions, cellular components, and biological processes (Figure 6C). KEGG pathway analysis further indicated that these targets are involved in phenylalanine metabolism, plant–pathogen interactions, and the biosynthesis of secondary metabolites (Figure 6D), suggesting a role for CsMYB82 in coordinating multiple stress‐related pathways. Motif analysis of the CsMYB82‐enriched peaks identified a conserved binding motif, AC‐III (ACCTAAC), along with several other enriched cis‐elements (Figure 6E).

### 2.7 CsMYB82 Directly Binds to and Activates *CsCAD4* Promoter

Lignin biosynthesis is known to be induced in tea plants as a defence mechanism against pathogen invasion (Kärkönen and Koutaniemi 2010; Zhao and Dixon 2011). Analysis of DAP‐seq data identified a *CAD* family gene (TEA000482), named *CsCAD4*, as a putative direct target of *CsMYB82*. A 766 bp *CsCAD4* promoter was amplified from the ‘ZC108’ cultivar based on the binding peak identified by DAP‐seq. Motif analysis revealed the presence of an AC‐III element (ACCTAAC) at 253 bp, along with three SMRE elements at 402 bp, 741 bp, and 759 bp, respectively (Figure S7A,B).

**FIGURE 7:**
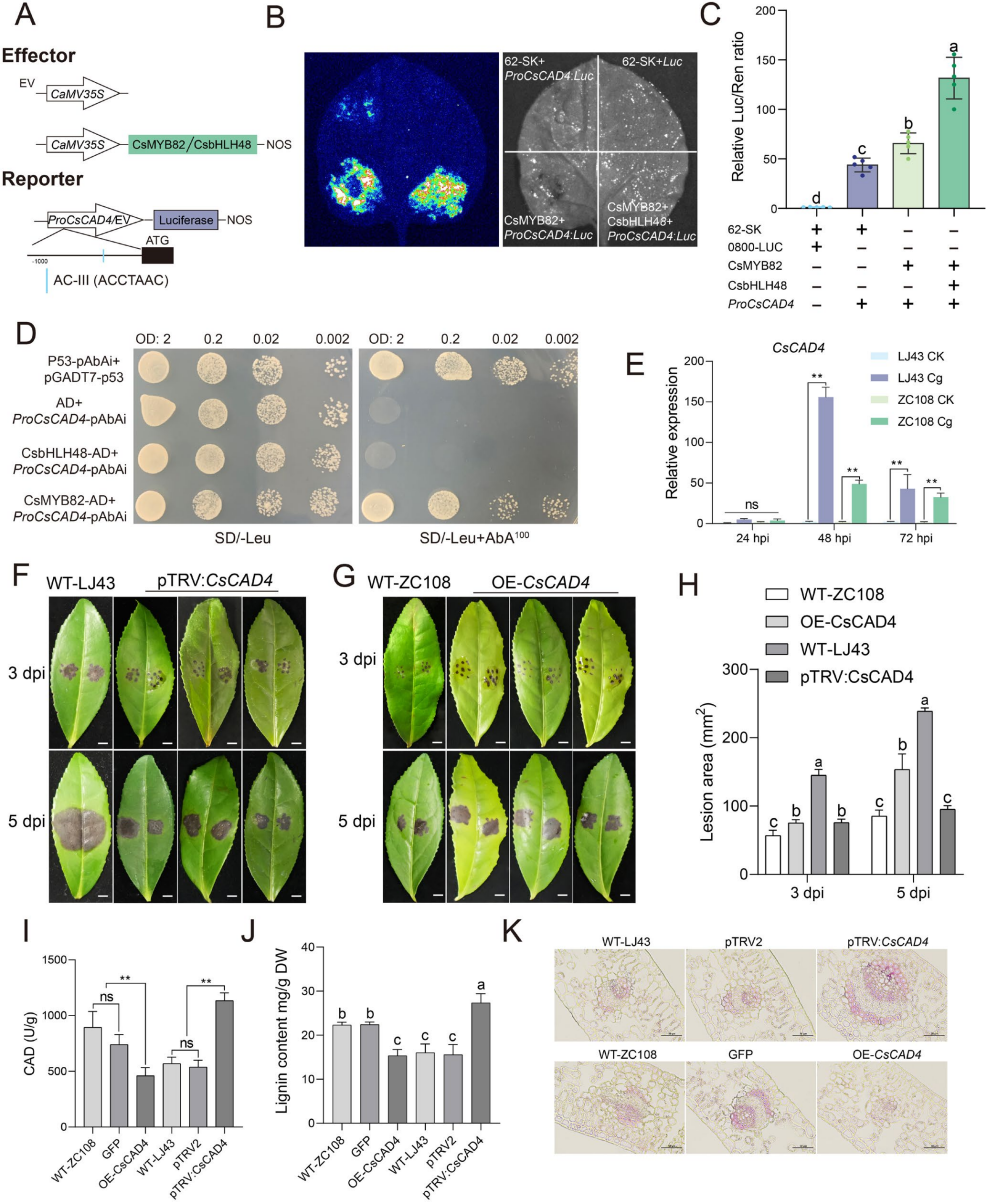
CsMYB82‐CsbHLH48 synergistically activate *CsCAD4* expression and the role of *CsCAD4* in tea anthracnose resistance. (A) Schematic diagram of the effector and reporter constructs for dual‐luciferase assay. (B) Luciferase (Luc) assay showing that CsMYB82 activates promoter activities of *CsCAD4* in *Nicotiana benthamiana* leaves. CsMYB82 co‐expressed with CsbHLH48 showed stronger activation of the *CsCAD4* promoter. The *ProCsCAD4*‐Luc + pGreenII 62‐SK and pGreenII 62‐SK + pGreenII 0800 Luc empty vector were co‐transformed for the control, respectively. (C) Relative LUC/REN expression in *Nicotiana benthamiana* leaves. CsMYB82 and CsbHLH48 co‐transformed activate promoter activities of CsCAD4. The DNA binding activity of *CsCAD4* by CsMYB82 was further enhanced after the formation of heterodimers between CsbHLH48 and CsMYB82 proteins. Values are presented as the means ± SD (*n* = 5). Different letters above the bars denote significant differences using a Tukey's multiple comparison test followed by one‐way ANOVA (*p* < 0.05). (D) Yeast One‐Hybrid assay indicated CsMYB82 specifically bind to *ProCsCAD4*, but CsbHLH48 can't bind. Yeast cultures were inoculated onto SD/−Leu media containing 100 ng mL^−1^ Aureobasidin A. Y1HGold[p53‐AbAi] co‐transformed with pGADT7‐p53 was used as positive control. Y1HGold[pAbAi‐*ProCsCAD4*] co‐transformed with pGADT7 recombinant plasmid was used as negative control and test group. (E) Expression analysis of *CsCAD4* by RT‐qPCR at 24, 48 and 72 h post inoculation (hpi) after *C. gloeosporioides* inoculation. Three independent replicates were conducted for each RT‐qPCR analysis. “ns” means no difference and asterisks indicate statistical significance using a Tukey's multiple comparison test followed by two‐way ANOVA (**p < 0.01). (F) Disease symptoms on pTRV: *CsCAD4* leaves after *C. gloeosporioides* inoculation at 3 and 5 days post inoculation (dpi) in ‘Longjing 43’ leaves. Scale bar = 2 cm. (G) Disease symptoms on OE‐*CsCAD4* leaves after *C. gloeosporioides* inoculation at 3 and 5 days post inoculation (dpi) in ‘Zhongcha 108’ leaves. Scale bar = 2 cm. (H) The lesion areas of pTRV: *CsCAD4*, OE‐*CsCAD4* and wild type (WT‐LJ43, WT‐ZC108) leaves at 3 and 5 days post inoculation (dpi). Values are presented as the means ± SD (*n* = 5). Different letters above the bars denote significant differences using a Tukey's multiple comparison test followed by two‐way ANOVA (*p* < 0.05). Measurements were performed at the indicated time points and comparisons were conducted within each time point. (I) Comparison of CAD activities pTRV: *CsCAD4*, OE‐*CsCAD4* and wild type (WT‐LJ43, WT‐ZC108) leaves after infiltrated leaves at 72 h. Values are presented as the means ± SD (*n* = 4). “ns” means no difference and asterisks indicate statistical significance using a Tukey's multiple comparison test followed by one‐way ANOVA (**p < 0.01). (J) Measurement of the total lignin content in pTRV: *CsCAD4*, OE‐*CsCAD4* and wild type (WT‐LJ43, WT‐ZC108) leaves after infiltration at 72 h. Values are presented as the means ± SD (*n* = 6). Different letters above the bars denote significant differences using a Tukey's multiple comparison test followed by one‐way ANOVA (*p* < 0.05). (K) Lignin accumulation of pTRV: *CsCAD4*, OE‐*CsCAD4* and control leaves through phloroglucinol staining on ‘Longjing 43’ and ‘Zhongcha 108’.

To validate the regulatory relationship between *CsMYB82* and *CsCAD4*, the dual‐luciferase reporter assay was performed using *CsCAD4* promoter: LUC as the reporter and 35S: CsMYB82 as the effector (Figure 7A). CCD imaging and quantitative analysis showed that CsMYB82 significantly increased LUC activity (~1.5‐fold), indicating positive regulation of CsCAD4 transcription (Figure 7B,C). To assess whether CsbHLH48 modulates this regulation, an additional dual‐luciferase (LUC) assay was conducted with co‐expression of CsMYB82 and CsbHLH48. Co‐expression significantly enhanced *ProCsCAD4* activation compared to CsMYB82 alone, suggesting that CsMYB82‐CsbHLH48 heterodimer formation promotes cooperative transcriptional activation (Figure 7C). The Y1H assay further confirmed that CsMYB82, but not CsbHLH48, directly binds to the *CsCAD4* promoter. Yeast harbouring CsMYB82 and *ProCsCAD4*‐pAbAi constructs grew on SD/−Leu medium containing 50 ng mL^−1^ AbA, whereas no growth was observed for CsbHLH48 (Figure 7D).

### 2.8 Characterisation of *CsCAD4* in Response to *C. gloeosporioides*

To investigate the expression dynamics and functional involvement of *CsCAD4* during anthracnose infection, we examined its transcriptional response and performed transient functional analyses in tea leaves. *CsCAD4* expression was further assessed following *C. gloeosporioides* infection. At 24 hpi, no significant difference in *CsCAD4* expression was observed between the two tea cultivars. By 48 hpi, transcript levels of *CsCAD4* increased sharply in both, with expression in ‘LJ43’ reaching 3.57‐fold higher than in ‘ZC108’. At 72 hpi, expression levels declined markedly in both cultivars (Figure 7E). To evaluate the functional role of *CsCAD4*, the gene was cloned from ‘ZC108’ and subjected to phylogenetic analysis, which showed that *CsCAD4* clustered with *AtCAD6* from *Arabidopsis thaliana* , suggesting potential functional conservation (Figure S7C). Transient OE‐*CsCAD4* and pTRV: *CsCAD4* in ‘ZC108’ and ‘LJ43’ leaves was confirmed (Figure S7D). Under *C. gloeosporioides* infection, OE‐*CsCAD4* leaves exhibited accelerated disease progression and enlarged lesion areas. In contrast, disease development was delayed and lesion areas were smaller in pTRV: *CsCAD4* leaves compared with the controls (Figure 7F–H). Consistently, CAD enzymatic activity was reduced in OE‐*CsCAD4* leaves but elevated in pTRV: *CsCAD4* leaves relative to the controls (Figure 7I). Moreover, lignin content was significantly decreased in OE‐*CsCAD4* leaves, whereas it was increased in pTRV: *CsCAD4* leaves, showing a pattern consistent with the observed changes in lignin accumulation (Figure 7J,K). In addition, the *CsCAD4* expression under genetic manipulation of *CsMYB82*, *CsMYB1* and *CsbHLH48* was evaluated. The results showed that CsMYB82 positively regulated *CsCAD4* expression (Figure S7E). By contrast, altering *CsbHLH48* expression had no detectable effect on *CsCAD4*, as *CsCAD4* transcript levels remained unchanged in both OE‐*CsbHLH48* and pTRV: *CsbHLH48* leaves compared with the respective controls (Figure S7F). Interestingly, in OE‐*CsMYB1* leaves, *CsCAD4* expression was significantly repressed, whereas no significant change in *CsCAD4* transcript levels was observed in pTRV: *CsMYB1* leaves relative to controls (Figure S7G).

### 2.9 Natural Variation in Lignin Accumulation and *CsMYB82* Expression Correlates With Anthracnose Resistance Across Tea Cultivars

**FIGURE 8:**
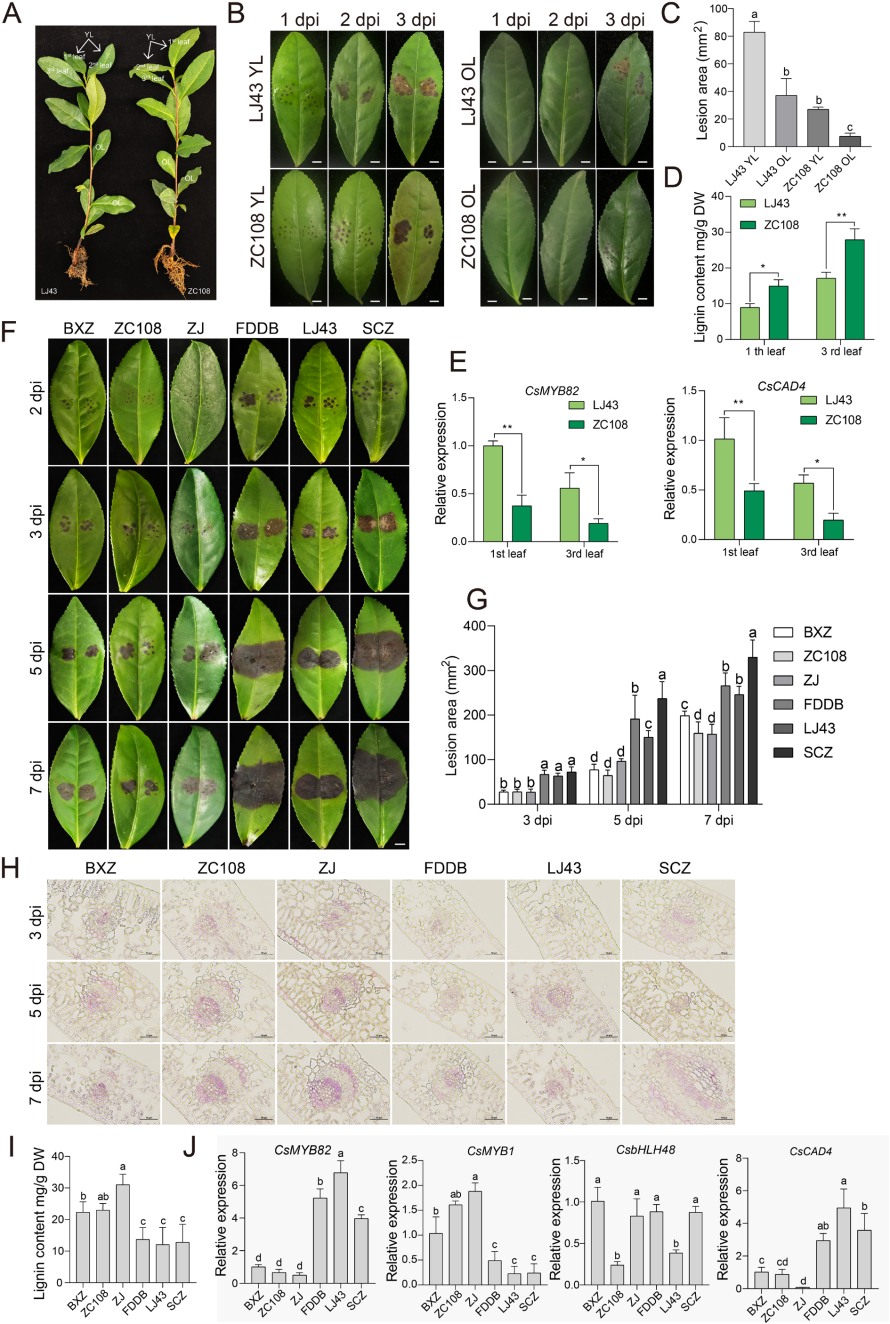
The characteristics of different resistant tea tree varieties after being infected by *C. gloeosporioides*. (A) Photograph of the leaves during development from ‘Longjing 43’ and ‘Zhongcha 108’. YL (Young leaf, the first to second leaf position), OL (Old leaf, the eighth to ninth leaf position). (B) Disease symptoms on tea plants leaves with different leaf positions after *C. gloeosporioides* inoculation at 1, 2, 3 days post inoculation (dpi). Scale bar = 2 cm. (C) The lesion areas of Young leaf (YL) and Old leaf (OL) in ‘Longjing 43’ and ‘Zhongcha 108’ at 3 day post inoculation (dpi). Values are presented as the means ± SD (*n* = 3). Different letters above the bars denote significant differences using a Tukey's multiple comparison test followed by one‐way ANOVA (*p* < 0.05). (D) Measurement of the total lignin content in the first and third leaf positions of ‘Longjing 43’ and ‘Zhongcha 108’ leaves. Values are presented as the means ± SD (*n* = 6) and asterisks indicate statistical significance (*p < 0.05, **p < 0.01). (E) Expression analysis of *CsMYB82* and *CsCAD4* by RT‐qPCR in the first and third leaf positions of ‘Longjing 43’ and ‘Zhongcha 108’ leaves. The RT‐qPCR data were presented as means ± SD values with three biological replicates and asterisks indicate statistical significance (*p < 0.05, **p < 0.01). (F) Disease symptom on tea plant leaves after *C. gloeosporioides* inoculation at 2, 3, 5 and 7 days post inoculation (dpi). Scale bar = 2 cm. BXZ, Bixiangzao; ZC108, Zhongcha 108; ZJ, Zijuan; FDDB, Fudingdabai; LJ43, Longjing 43; SCZ, Shuchazao. (G) The lesion areas of six tea cultivars at 3, 5 and 7 days post inoculation (dpi). Values are presented as the means ± SD (*n* = 6). Different letters above the bars denote significant differences using a Tukey's multiple comparison test followed by two‐way ANOVA (*p* < 0.05). Measurements were performed at the indicated time points and comparisons were conducted within each time point. (H) Lignin accumulation through phloroglucinol staining at 3, 5 and 7 days post inoculation (dpi) on six tea cultivars. (I) Measurement of the total lignin content in six tea cultivars leaves under natural conditions. Values are presented as the means ± SD (*n* = 5). Different letters above the bars denote significant differences using a Tukey's multiple comparison test followed by one‐way ANOVA (*p* < 0.05). (J) Expression levels of *CsMYB82*, *CsMYB1*, *CsbHLH48* and *CsCAD4* in the second leaf position of different tea cultivars. Values are presented as the means ± SD (*n* = 3). Different letters above the bars denote significant differences using a Tukey's multiple comparison test followed by one‐way ANOVA (*p* < 0.05).

Given that lignin accumulation varies with tissue type and developmental stage, we first examined the relationship between leaf position, lignin content, disease severity, and gene expression in tea cultivars with contrasting anthracnose resistance. Following *C. gloeosporioides* inoculation, the susceptible cultivar ‘LJ43’ exhibited earlier onset and more severe disease symptoms than the resistant cultivar ‘ZC108’. Within each cultivar, younger leaves were consistently more severely affected than older leaves (Figure 8A–C). Under natural growth conditions, lignin content measured at the same leaf position was significantly higher in resistant cultivars than in susceptible ones (Figure 8D). To further explore the association between gene expression and disease severity, the transcript levels of *CsMYB82* and *CsCAD4* were quantified in different leaf positions. Both genes showed significantly higher expression in first‐position leaves than in third‐position leaves in both ‘ZC108’ and ‘LJ43’ (Figure 8E), consistent with the greater disease severity observed in younger leaves.

**FIGURE 9:**
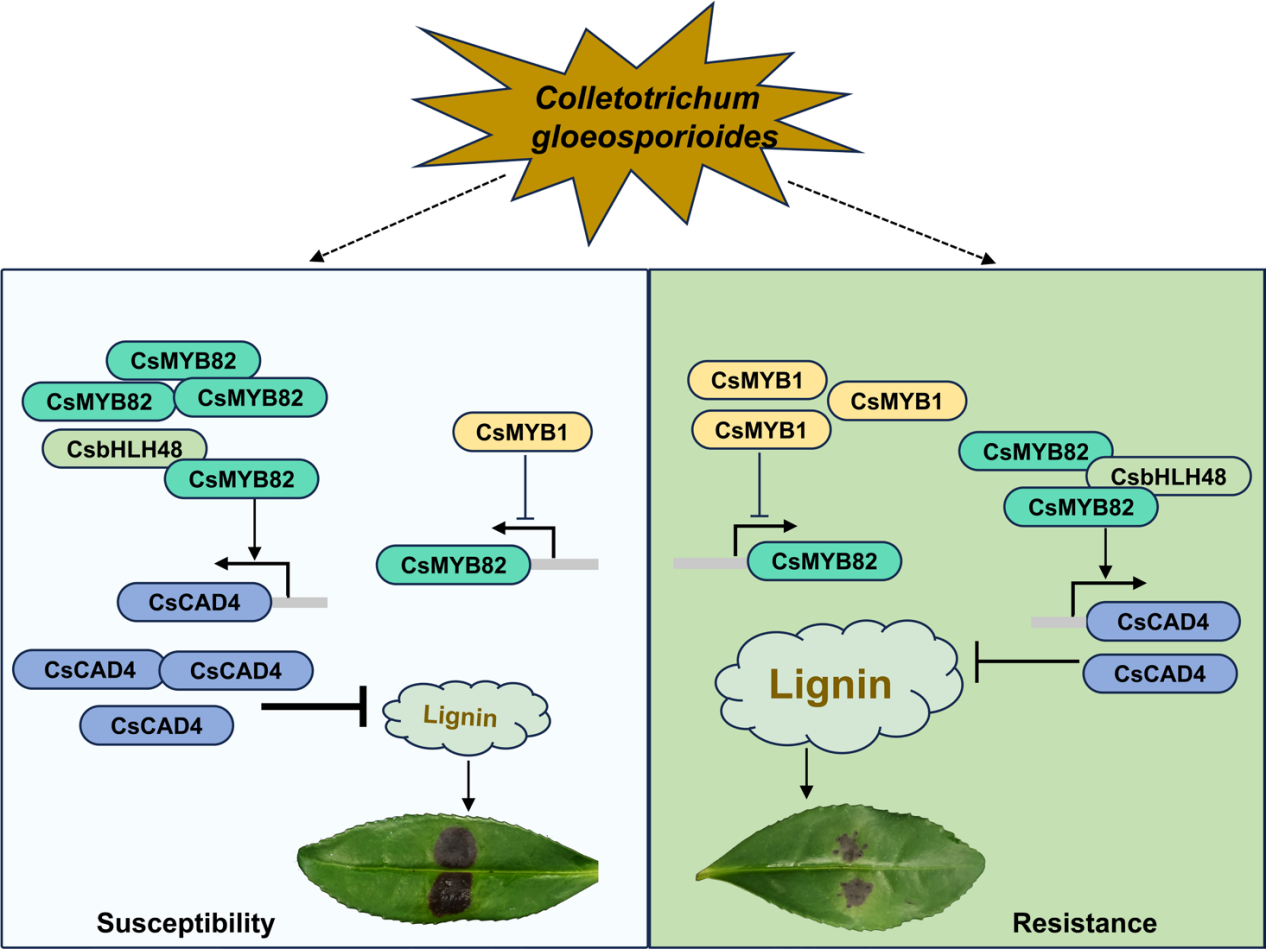
A working model of *CsMYB82* mediated resistance to *C. gloeosporioides* in tea plants. Upon *C. gloeosporioides* infection, CsMYB1 is induced and represses CsMYB82 transcription. CsMYB82 directly activates the downstream target CsCAD4 and this promoter activation is enhanced by its interaction with CsbHLH48, which functions as a context‐dependent co‐regulator. Suppression of CsMYB82 is associated with increased lignin accumulation and enhanced resistance, whereas elevated CsMYB82/CsCAD4 is linked to reduced lignification and higher susceptibility. Natural variation across six tea cultivars supports the generality of this module: Resistant genotypes show higher lignin and higher CsMYB1 but lower CsMYB82/CsCAD4, while susceptible genotypes show the opposite trend; CsbHLH48 displays limited cultivar‐level variation. Arrows indicate activation, blunt‐ended lines indicate repression.

We next compared anthracnose resistance among six representative tea cultivars to evaluate whether cultivar‐level variation was associated with lignin accumulation and gene expression patterns. Upon *C. gloeosporioides* inoculation, ‘LJ43’, ‘Fuding Dabai (FDDB)’, and ‘Shuchazao (SCZ)’ developed more severe disease symptoms, whereas ‘ZC108’, ‘Bixiangzao (BXZ)’, and ‘Zijuan (ZJ)’ exhibited markedly milder symptoms (Figure 8F). Quantitative analysis showed that lesion areas increased over time in all cultivars, but remained significantly smaller in resistant cultivars at 3, 5, and 7 dpi (Figure 8G). Phloroglucinol‐HCl staining further revealed higher lignin accumulation in resistant cultivars following infection (Figure 8H). *CsMYB1* was upregulated at 2 and 3 dpi in all cultivars; at 5 dpi, *CsMYB1* remained significantly induced in ‘BXZ’, ‘ZC108’, and ‘LJ43’, but showed no significant change in ‘ZJ’, ‘SCZ’ and ‘FDDB’. *CsMYB82* displayed a clear divergence among cultivars: it was repressed after infection in ‘BXZ’, ‘ZC108’, and ‘ZJ’, whereas it was induced in ‘LJ43’, ‘SCZ’ and ‘FDDB’. *CsbHLH48* was consistently downregulated at 2, 3 and 5 dpi in ‘BXZ’ and ‘ZJ’, showed no significant change in ‘ZC108’, and exhibited a delayed induction in ‘LJ43’ (no significant difference at 2 and 3 dpi but significant upregulation at 5 dpi). In ‘FDDB’, *CsbHLH48* was suppressed at 2 and 3 dpi but became upregulated at 5 dpi. *CsCAD4* showed a broadly conserved early response: it was significantly upregulated at 2 dpi in all six cultivars. At 3 dpi, *CsCAD4* showed no significant change in ‘BXZ’ and ‘ZC108’ but was significantly induced in ‘ZJ’, ‘LJ43’, ‘SCZ’ and ‘FDDB’. At 5 dpi, *CsCAD4* remained upregulated in ‘BXZ’ and ‘ZJ’, whereas no significant difference was detected in the other four cultivars (Figure S8A). Similar expression patterns were also observed under natural conditions, with *CsMYB82* remaining expressed at lower levels in resistant cultivars than in susceptible ones. In addition, *CsCAD4* exhibited an expression pattern comparable to that of *CsMYB82*, showing higher transcript levels in susceptible cultivars. In contrast, *CsMYB1* displayed an opposite trend, with higher expression in resistant cultivars, whereas *CsbHLH48* expression did not show obvious differences between resistant and susceptible cultivars (Figure 8J). Furthermore, disease‐resistant varieties also have high levels of lignin under natural conditions (Figure 8I). In addition, lignin accumulation differed markedly among the six tea cultivars after *C. gloeosporioides* infection. Specifically, ‘BXZ’, ‘ZC108’ and ‘ZJ’ consistently exhibited significantly higher lignin contents at 2, 3, and 5 dpi compared with ‘FDDB’, ‘LJ43’ and ‘SCZ’ (Figure S8B). Overall, variation in anthracnose resistance among tea cultivars coincided with differences in lignin accumulation and the expression profiles of CsMYB82, CsCAD4, and CsMYB1, suggesting a coordinated association between these molecular traits and disease outcomes.

## 3 Discussion

Lignin deposition is widely recognised as an effective cell‐wall‐based defence that can restrict fungal ingress by reinforcing the physical barrier and limiting pathogen spread (Park et al. 2019; Ren et al. 2020). In this study, we combined cultivar comparisons, overexpression and VIGS‐function assays, protein–protein interaction analyses, and DAP‐seq‐guided target identification to reveal a lignin‐associated transcriptional module centred on CsMYB82 during *C. gloeosporioides* infection. Our results support a working model in which the upstream factor CsMYB1 represses *CsMYB82*, while CsMYB82 cooperates with CsbHLH48 to enhance activation of the downstream target *CsCAD4*, thereby influencing lignin accumulation and anthracnose outcomes.

CADs typically catalyse the final step of monolignol biosynthesis and are often induced after infection to promote localised lignification. In Arabidopsis, nine CAD genes (AtCAD1‐9) have been identified: AtCAD4/5 show high activity toward cinnamaldehyde, whereas AtCAD7/8 (together with StuCAD6 in potato) contribute little to lignification (Costa et al. 2003; Kim et al. 2004, 2007; Li et al. 2019). Our phylogenetic analysis placed CsCAD4 within the AtCAD6‐related clade (Figure S7C), which is consistent with the possibility that CsCAD4 may differ from the best‐characterised lignification‐associated CADs. CsCAD4 was strongly induced by *C. gloeosporioides* in susceptible cultivars and repressed in resistant cultivars (Figure 7E). Furthermore, its transient overexpression increased susceptibility, whereas pTRV: *CsCAD4* delayed disease progression (Figure 7F–H), suggesting that CsCAD4 may function as a non‐canonical, potentially pathogen‐exploitable factor rather than a straightforward lignification‐promoting enzyme. And in our transient perturbation assays, CsCAD4 consistently correlated with lignification‐related outputs: transient overexpression was accompanied by reduced total CAD activity and decreased total lignin accumulation, whereas transient silencing showed the opposite trend (Figure 7I–K). While these results do not by themselves establish the precise biochemical mechanism of CsCAD4, they support the view that CsCAD4 is unlikely to function as a dominant, straightforward lignification‐promoting CAD under our tested conditions. The observed trends could reflect pathway‐level regulation, interactions among CAD paralogs, substrate availability, flux partitioning, and enzyme or post‐transcriptional regulation. Future work combining targeted metabolomics of lignin‐pathway intermediates and monolignol derivatives with lignin compositional analyses, together with a broader assessment of other CsCAD family members, will be important to clarify the biochemical basis of cultivar‐dependent lignin differences and resistance‐associated lignification.

MYB genes participate in diverse regulatory processes in plants, including defence responses. In Arabidopsis, overexpression of AtMYB58, AtMYB63, and AtMYB15 activates the expression of lignin biosynthesis‐related genes such as PAL, C4H, 4CL, and CAD, thereby promoting cellular lignification; accordingly, lignin deposition and disease resistance are reduced in the myb15 mutant (Kim et al. 2020). Overexpression of *CmMYB19* in chrysanthemum enhances lignin accumulation and consequently restricts the reproduction of *Macrosephoniella sanborni* (Wang, Sheng, et al. 2017). In our study, CsMYB82 was identified as a pathogen‐responsive R2R3‐MYB transcription factor whose expression was induced upon *C. gloeosporioides* infection in an anthracnose‐susceptible tea cultivar (Figure 2A). Functional analyses based on transient manipulation showed that overexpression of CsMYB82 was associated with increased disease susceptibility and reduced lignin accumulation, whereas silencing of CsMYB82 correlated with enhanced resistance and elevated lignin levels (Figures 2D–H and 3A,D,E).

Mechanistically, CsMYB82 functions within an integrated regulatory module connecting an upstream repressor, a cooperative partner, and a downstream effector. The upstream factor CsMYB1 is strongly induced upon infection and directly represses CsMYB82 transcription by binding to its promoter, thereby promoting resistance (Figures 4 and S5E,F). Similar inhibitory mechanisms have been documented in other systems: the JAZ1‐RcMYB84 complex binds the CAACTG motif in the *RcMYB123* promoter to suppress transcription (Ren et al. 2020), and CmMYB012 directly interacts with an AACATT element in the *CmFNS* promoter to negatively regulate thermotolerance (Zhou et al. 2021). At the downstream level, CsMYB82 targets *CsCAD4* by binding to its promoter and activating its transcription, and this activation is further potentiated by physical interaction with CsbHLH48, which acts as a co‐regulator to amplify CsMYB82‐dependent promoter activation (Figures 5A–D and 7B–D). Functional analyses showed that manipulating CsbHLH48 alone—via transient overexpression or VIGS—did not significantly affect anthracnose severity or lignin accumulation in tea leaves (Figure 5E–G,J), suggesting that it is unlikely to function as an independent regulator of lignin‐mediated defence. Instead, its contribution may be context‐dependent, requiring CsMYB82 and/or other cofactors. Although CsbHLH48 exhibited higher basal expression in the third leaf position of the resistant cultivar under natural conditions (Figure 5H), this difference did not translate into measurable lignin or resistance changes when CsbHLH48 was manipulated alone. Similarly, in lily plants, LlETO1 does not directly bind to the LlMBF1c promoter; instead, it cooperates with LlERF092 to promote LlMBF1c promoter activation, thereby regulating lily heat tolerance (Xiang et al. 2025). Our previous study also showed that CsbHLH62 forms a complex with CsNAC17, which enhances CsNAC17 binding to the *CsRPM1* promoter, thereby promoting tea plant resistance to the anthracnose pathogen (Han et al. 2025).

DAP‐seq provided a genome‐wide view of CsMYB82 DNA association and suggested that CsMYB82 may regulate stress programs beyond lignin biosynthesis. Enrichment of CsMYB82 targets in phenylalanine metabolism, plant‐pathogen interaction, and secondary metabolite pathways indicates a broad regulatory potential during infection (Figure 6C,D). Thus, although our data support a MYB‐bHLH‐CAD module in anthracnose responses, CsMYB82 (and CsCAD4) may not act exclusively in lignin formation, as MYB factors often coordinate multiple phenylpropanoid branches, including flavonoid biosynthesis and salicylic acid‐related signalling (Wang, Ran, et al. 2017; Camargo‐Ramírez et al. 2018; Guo et al. 2024). These DAP‐seq resources therefore provide a foundation for future dissection of additional CsMYB82‐regulated defence metabolites and hormone‐linked pathways that may influence susceptibility. Comprehensive metabolomic profiling and pathway‐level flux analyses will be required in future studies to determine whether CsMYB82‐ and CsCAD4‐mediated regulation extends to flavonoid, SA, or other defence‐related metabolic pathways and how these interactions collectively shape anthracnose resistance.

Beyond mechanistic dissection, the association between this regulatory module and natural resistance variation enhances the relevance of our findings. Younger leaves showed higher expression of CsMYB82 and CsCAD4 and exhibited greater disease severity, consistent with their enhanced susceptibility (Figure 8E). Across tea cultivars, the resistance phenotype was associated with a coordinated transcriptional pattern characterised by lower CsMYB82/CsCAD4 but higher CsMYB1 expression and higher lignin accumulation (Figures 8I,J and S8). The regulatory model proposed in this study reveals cultivar‐dependent variation in anthracnose resistance.

In addition, several functional conclusions rely on transient manipulation in tea leaves and heterologous assays in tobacco, owing to the lack of stable genetic transformation methods in tea plant. Notably, the EV treatments themselves affected lignin‐pathway transcripts: pTRV2‐EV was largely similar to WT (except Cs4CL and CsHCT), whereas pCAMBIA2300A‐EV caused broad upregulation relative to WT (Figure S3), indicating a confounding agroinfiltration/vector effect (Pitzschke 2013; Garcia and Steinbrenner 2023). Accordingly, we interpret our results primarily based on comparisons to matched EV controls and concordant evidence from phenotypes, lignin measurements, and promoter/interaction assays, and future work should incorporate additional controls (e.g., mock infiltration) and stable/genome‐editing validation when feasible.

In conclusion, our findings reveal a regulatory model in which low expression of CsMYB1 in the susceptible tea cultivar fails to repress *CsMYB82*, thus CsMYB82 activates *CsCAD4*. In contrast, the resistant tea cultivar exhibits higher *CsMYB1* and lower *CsMYB82*/*CsCAD4* expression, leading to increased lignin content and enhanced pathogen resistance (Figure 9). This framework is consistent with natural variation across six tea cultivars. Future work integrating metabolomics, stable/genome‐editing validation, and greenhouse/field trials across diverse germplasm will be needed to test the broader relevance and breeding utility of this module.

## 4 Materials and Methods

### 4.1 Plant Materials and Growth Conditions

The two‐year‐old resistant tea plant cultivar “Zhongcha 108 (ZC108)” and the susceptible tea cultivar “Longjing 43 (LJ43)”, along with transgenic and wild‐type tobacco (*Nicotiana benthamiana*) seedlings derived from plant tissue culture, were maintained under controlled environmental conditions: a constant temperature of 25°C, 85% relative humidity, and a 16‐h light/8‐h dark photoperiod. An additional ten cultivars of tea plants, all two‐year‐old, were sourced from Nanjing Yarun Co. Ltd. (Nanjing, Jiangsu, China) and maintained under controlled conditions in a light‐growth chamber.

### 4.2 Bioinformatic Analyses and Subcellular Localisation

Phylogenetic analysis was conducted using MEGA5.0. Protein conserved domains were identified through dual‐platform analysis using SMART (http://smart.embl‐heidelberg.de) and NCBI's CDD (https://www.ncbi.nlm.nih.gov/Structure/cdd/wrpsb.cgi). Chromosomal mapping of the gene was conducted using the MG2C online platform (http://mg2c.iask.in/mg2c_v2.1/) with the shuchazao V2. Genome of TPIA (https://tpia.teaplants.cn/). The coding sequence (CDS) of *CsMYB82*, *CsbHLH48*, *CsMYB1* and *CsCAD4* lacking a stop codon, were PCR‐amplified from “ZC108” using specific primers and subsequently inserted into the pCAMBIA2300 GFP vector. The recombinant vectors, *CsMYB82*‐GFP, *CsbHLH48*‐GFP, *CsMYB1*‐GFP, *CsCAD4*‐GFP, and unmodified pCAMBIA2300 GFP were individually transformed into *Agrobacterium tumefaciens* GV3101, followed by transient expression in *Nicotiana benthamiana* epidermal cells through syringe infiltration (Fan et al. 2017). The fluorescence signal was measured at 488 nm by utilising a laser confocal microscope (ZEISS LSM 800, Germany). The specific primers employed in these constructions are enumerated in Table S1.

### 4.3 Generation of Transgenic Tobacco

The subcellular localisation recombinant plasmids were employed for the genetic transformation of tobacco, following a previously established protocol (Sparkes et al. 2006). The expression of *CsMYB82* genes was assessed using RT‐qPCR. And the non‐transgenic wild‐type tobacco was utilised as the negative control in this study. The specific primers employed in these experiments are listed in Table S2.

### 4.4 Transient Overexpression Assays on *C. sinensis* Leaves

The *A. tumefaciens* GV3101 strain carrying recombinant plasmid was cultured at 28°C with shaking at 200 rpm until it reached an optical density of OD_600_ = 1.0. The *A. tumefaciens* cells were harvested through centrifugation, and subsequently resuspended in infiltration buffer according to the previously described protocol (Shui et al. 2023). The infiltration buffer was injected into petioles using a needle syringe. Leaf position was defined by counting from the shoot apex downward, and only fully expanded leaves were used. For gene expression analysis and lignin content measurement, agroinfiltration and sampling were performed on the second fully expanded leaf of ‘ZC108’ or ‘LJ43’. For infection assays after transient transformation, pathogen inoculation was performed on the third fully expanded leaf, and all corresponding controls were treated from the same leaf position. Within each assay, all treatments and controls were collected from the same leaf position. Subsequently, the plants were cultivated under controlled conditions at a temperature of 24°C and a relative humidity range of 60%–70% in low‐intensity light. The wild type and empty vectors were utilised as control groups.

### 4.5 VIGS Analysis on *C. sinensis* Leaves

The 348, 399 and 213 bp cDNA fragment of *CsMYB82*, *CsMYB1* and *CsCAD4* was successfully incorporated into the pTRV2 plasmid, respectively, followed by transformation of the resulting recombinant vector into *A. tumefaciens* GV3101 according to the previously described protocol. The recombinant plasmid‐carrying *A. tumefaciens* GV3101 was cultured at 28°C with shaking at 200 rpm until it reached an optical density of OD_600_ = 1.0. The *A. tumefaciens* GV3101 carrying the target fragment and pTRV1 were combined in a 1:1 ratio, and injected into ‘ZC108’ and ‘LJ43’ leaves as both the experimental group and negative control group. The leaves without any strain injection were used as the control group. For each treatment, each biological replicate consisted of at least 3 independently infiltrated leaf from a different individual plant.

### 4.6 Pathogen Infection, Histochemical Staining, Determination of CAD Enzyme Activity and Lignin Content

The conidial concentration of *C. gloeosporioides* determined using a haemocytometer was adjusted to 1 × 10^6^ ML^−1^ for inoculation. For infection assays following transient transformation in tea plants, inoculation was performed on the third fully expanded leaf of ‘ZC108’ and ‘LJ43’, and all corresponding controls were treated and assessed using the same leaf position. The tea plant leaves, transgenic tobacco, and wild‐type tobacco were inoculated with *C. gloeosporioides* according to established protocols (Shen et al. 2001; Jeyaraj et al. 2021).

As previously mentioned, trypan blue staining was conducted to visualise fungal development in the second leaves, and DAB staining was performed to assess the accumulation of H_2_O_2_ (Wang et al. 2018; Han, Yin, et al. 2021). The cross sections of the second leaves were stained with phloroglucinol to facilitate the observation of total lignin deposition (Xu et al. 2011). The lignin content was determined by kit method provided by the manufacturer of Sangon Biotech in the second leaves (Shanghai, China) (Yuan et al. 2023). The histochemical staining was examined under Olympus IX73 stereo and light microscope (Olympus IX73, Japan). The lesion areas were calculated using Image J (NIH, Bethesda, MD, USA). CAD activity was determined using a commercial assay kit (Catalogue No. AKSU053U; Boxbio) according to the manufacturer's instructions. Fungal biomass was quantified by qPCR as the ratio of fungal genomic DNA to host genomic DNA in infected leaf tissues. Total DNA was extracted at each time point, and fungal abundance was calculated by normalising a fungal‐specific marker to a plant reference gene using the $2^{-∆C_{t}}$ method. For OE‐*CsMYB82*, leaf tissues from multiple independent transgenic lines were pooled in equal amounts to form biological replicates before DNA extraction.

### 4.7 Yeast Two‐Hybrid Screening and Assay

The transcriptional autoactivation of *CsMYB82* was evaluated using the protocols outlined in the Matchmaker Gold Yeast Two Hybrid System User Manual (PT4084‐1) provided by Clontech. The positive control pGBKT7‐53 and the negative control pGBKT7‐Lam were utilised in this study.

The yeast library preserved in the laboratory was utilised. The User Manual for the MatchmakerGold yeast Two Hybrid library Screening System was consulted to identify the insertion fragment, which was subsequently subjected to sequencing. The suitable candidate prey was chosen from the screened positive clones, and its CDS was inserted into the pGADT7 (AD) vector. Furthermore, we verified the interaction between CsMYB82 and candidate prey proteins in yeast according to a previous study (Cheng et al. 2023; Liu, Wang, Liu, et al. 2023).

### 4.8 Bimolecular Fluorescence Complementation Assay

The full‐length CDSs of *CsMYB82* and *CsbHLH48*, which lack stop codons, were cloned into pCV‐nYFP and pCV‐cYFP vectors to generate fusion constructs nYFP‐CsMYB82 and cYFP‐CsbHLH48. The *Agrobacterium*‐mediated transformation method was utilised for transient expression of these constructs, along with the mCherry marker, in *N. benthamiana* leaves. The fluorescence signal was observed using confocal laser scanning microscopy (ZEISS LSM 800, Germany) following a 48‐h incubation period (Zhang, Tong, et al. 2023).

### 4.9 Split‐Luciferase (LUC) Complementation Assay

The full‐length CDSs of *CsMYB82* and *CsbHLH48* were cloned into the nLuc and cLuc vectors, respectively. The resulting constructs (nluc‐CsMYB82 and cluc‐CsbHLH48) were co‐transformed into *A. tumefaciens* GV3101 for a 2‐day culture at 28°C, followed by co‐injection into tobacco leaves that were 4–5 weeks old for Split‐LUC analysis. The *Agrobacterium* GV3101 strains containing the fusion plasmid nLUC‐CsMYB82 and cLuc, nLUC and cLUC‐CsbHLH48, nLUC and cLuc were mixed (nLUC‐CsMYB82: cLuc = 1:1; nLUC: cLUC‐CsbHLH48 = 1:1; nLUC: cLUC = 1:1, OD_600_ = 0.8) as control. After a 2‐day period of tobacco growth, the fluorescence signals were collected using a CCD instrument (Lumizone Pylon 2048B).

### 4.10 Co‐Immunoprecipitation Assay

The *CsMYB82* and *CsbHLH48* were cloned into the vectors pCAMBIA2300‐GFP (CsMYB82‐GFP) and pCAMBIA2300‐3 × FLAG (CsbHLH48‐FLAG), respectively, for Co‐immunoprecipitation experiments. After *Agrobacterium* GV3101 transformation, the tobacco leaves were infiltrated and subsequently rapidly frozen after approximately 2 days. Total protein was then extracted according to the manufacturer's instructions (YEASEN, Shanghai, China). The Co‐immunoprecipitation assay was conducted in accordance with the previously established protocol (Guo et al. 2023).

### 4.11 Yeast One‐Hybrid Screening and Assay

The Y1HGold‐pAbAi Yeast One‐Hybrid Library Screening Kit (Coolaber, Beijing, China) was utilised for the screening library. The pAbAi vector was employed for the construction of a recombinant plasmid, namely pAbAi‐ProCsMYB82, which encompasses the promoter sequence of CsMYB82 (1712 bp). A positive control was established using the pGADT7‐p53 transformed Y1HGold [p53‐AbAi] yeast strain, while a negative control was set up using the pGADT7 transformed Y1HGold [pAbAi‐*ProCsMYB82*] yeast strain. The promoter sequence of *CsCAD4*, a downstream target gene, was subsequently cloned into the pAbAi vector to generate pAbAi‐*ProCsCAD4* as bait and *CsMYB82* CDS was inserted into the pGADT7 vector to construct AD‐CsMYB82 as prey. The unaltered pGADT7 vector‐transformed bait strain was utilised as a negative control.

### 4.12 Dual‐Luciferase Assay

The CDS of *CsMYB82* and *CsbHLH48* were cloned into the pGreenII 62‐SK transient expression vector as effectors. The *ProCsCAD4* was inserted into the pGreenII0800‐LUC transient expression vector as reporter. The reporter and effector plasmid were co‐infiltrated into tobacco leaves using *A. tumefaciens* GV3101 (pSoup), employing the following combinations: 62‐SK‐CsMYB82 + *ProCsCAD4*‐LUC, 62‐SK‐CsMYB82 + 62‐SK‐CsbHLH48 + *ProCsCAD4*‐LUC and 62‐SK‐CsbHLH48 + *ProCsCAD4*‐LUC. The empty pGreenII 62‐SK and pGreenII 0800‐LUC vectors were utilised as experimental controls, and the luciferase image was acquired utilising a CCD instrument (PIXIS 1024B). The LUC activities were evaluated using the Dual‐Luciferase Reporter Assay System in accordance with the manufacturer's instructions (Yeasen, Shanghai, China).

### 4.13 GUS Staining and Activity

The *CsMYB1* CDS and *CsMYB82* promoter (1712 bp) were cloned into 35S‐GFP and pCAMBIA1300‐GUS (No Pro35S) vectors as effector and reporter plasmids, respectively. The constructs were subsequently transformed into *A. tumefaciens* GV3101. The experimental group was composed of 35S:CsMYB1 + *ProCsMYB82*: GUS, while the control group consisted of 35S‐GFP + *ProCsMYB82*‐GUS. The effector plasmid was combined with an equal volume of *Agrobacterium* harbouring the reporter plasmid in both experimental groups. After 48 h of tobacco leaf injection, GUS staining and activity were conducted in accordance with the manufacturer's instructions, followed by subsequent protein extraction (SL7160, SL7161; Coolaber) (Wang et al. 2023).

### 4.14 DNA Affinity Purification Sequencing

The DNA Affinity Purification Sequencing (DAP‐Seq) test method was performed by the previously established protocol (Bartlett et al. 2017). The genomic DNA was extracted from the tender leaves of ‘ZC108’, and a DNA library was constructed using the NGS0602‐MICH TLX DNA‐Seq Kit. Subsequently, *CsMYB82* underwent in vitro protein expression, and the DNA fragments that interacted with *CsMYB82* were selectively enriched using magnetic beads through a combination of protein and library mixing. After performing DNA washing to eliminate non‐specific binding, the complex underwent purification using the PE150 method on Illumina NovaSeq 6000 and was subsequently subjected to sequencing. The alignment of the acquired data to the tea plant genome was performed utilising BWA‐MEM (Vasimuddin et al. 2019). The Model‐based analysis of ChIP‐Seq (MACS) software version 2.2.7.1 was utilised for peak detection in order to identify peaks that are enriched relative to the Input sample (Zhang et al. 2008). Additionally, MEME‐ChIP software version 5.0.5 was utilised for the analysis of characteristic motifs within the identified peak regions, while Homer software version v4.10 was employed for the annotation of these peaks (Heinz et al. 2010; Machanick and Bailey 2011). The GO enrichment analysis was conducted using the g: Profiler gene annotation website, while the KEGG enrichment analysis was performed on the online platform provided by https://www.genome.jp/kegg/.

### 4.15 Real‐Time Quantitative PCR (RT‐qPCR)

The total RNA of tea leaves under different treatments was extracted using the Accurate Biology Complex Plant RNA Kit (Accurate Biology, Hunan, China), following the manufacturer's protocol. The HiScript III RT SuperMix for qPCR with gDNA wiper (Vazyme, Nanjing, China) was employed to synthesise the first‐strand cDNA for RT‐qPCR analysis. The RT‐qPCR analysis was conducted using the ChanQ SYBR‐qPCR Master Mix (Vazyme, Nanjing, China). *β*‐Actin was served as a reference gene. Relative transcript levels were calculated using the $2^{-∆∆C_{t}}$ method. The RT‐qPCR tests were conducted using the QuantStudio 5 real‐time PCR detection system (ABI, USA). Specific primers used in these constructions are listed in Table S2. Representative lignin biosynthesis‐related genes were selected based on previously published studies in tea plants, focusing on genes that have been annotated and reported to participate in lignin biosynthesis or the phenylpropanoid pathway (Wang et al. 2019; Zhang, Zhang, et al. 2023). The experiments were conducted in three separate repetitions.

### 4.16 Accession Numbers

Sequence data in this article can be found in the Tea plant Genome Annotation Project Database or GenBank data libraries under the following accession numbers: CsMYB82(TEA023420), CsbHLH48(TEA003290), CsMYB1(TEA008298), CsCAD4(TEA000482), AtMYB82(AF048841), NtMYB82(XP_016450413), VvMYB82(XP_002282342).

## Author Contributions

R.H., Y.W. and Z.X. conducted the experiments. R.H., Y.W. and W.W. analysed the data. R.H. wrote the manuscript. C.M., J.Z., Z.Z. and W.Y. revised the manuscript. S.L., Y.H.W., J.Z., X.C. and Q.H. supervised the project. S.M. and X.L. conceived the project and acquired funding.

## Funding

This work was supported by the National Key Research and Development Program of China, 2022YFD1200505, Jiangsu Provincial Key Research and Development Program, BE2023364, National Program for SRT, X2025103070018, National Natural Science Foundation of China, 32172628, Science and Technology Plan Program of Wen County, 2024CX001, 2023‐X.QKJ‐02.

## Conflicts of Interest

The authors declare no conflicts of interest.

## Supporting information


**Figure S1:** Bioinformatic analyses of CsMYB82. (A) Chromosome location of *CsMYB82*. *CsMYB82* is located on chromosome 6 with two SANTs domain. (B) Protein sequence alignment of conserved domain of CsMYB82. At, *Arabidopsis thaliana* ; Nt, *Nicotiana tabacum* ; Vv, *Vitis vinifera* .
**Figure S2:** CsMYB82 phylogenetic analysis, transcriptional activation activity and identification of CsMYB82 transgenic leaves. (A) Phylogenetic analysis of CsMYB82 with the homologous genes in other species. (B) CsMYB82 transactivation assay in yeast. Co‐transformation of AD‐T with BD‐p53 or BD‐Lam into yeast cells was used as positive (Po) or negative controls (Ne), respectively. SD − Trp/X, SD − Trp/X‐α‐Gal; SD − Trp/X/A, SD − Trp/X‐α‐Gal/aureobasidin A. (C) The OE‐*CsMYB82* and pTRV: *CsMYB82* constructs. (D) Quantitative analysis of *CsMYB82* overexpression lines (L1, L2, L3, L4, L5, L6, L7, L8) and wild type (WT). The RT‐qPCR data were presented as means ± SD values with three biological replicates. Asterisks indicate statistical significance (**p < 0.01). (E) Petiole injection. The second leaf position was selected for the experiment. (F, G) Confirmation of Virus‐induced gene silencing (VIGS) and OE‐*CsMYB82* leaves by RT‐qPCR analysis. #1, #2, #3, #4, #5 and #6 were referred to the distinct pTRV: *CsMYB82* leaves in ‘Longjing 43’. WT(Wild‐type) and pTRV2 as controls. OE#1—OE#11 were referred to the distinct OE‐*CsMYB82* leaves in ‘Zhongcha 108’. WT(Wild‐type) and empty vector (EV) as controls. The RT‐qPCR data were presented as means ± SD values with three biological replicates. “ns” means no difference and asterisks indicate statistical significance (*p < 0.05, **p < 0.01). (H) Lignin accumulation through phloroglucinol staining in OE‐*CsMYB82* leaves. Scale bar = 100 μm.
**Figure S3:** Gene expression analysis in lignin synthesis pathway in pTRV: *CsMYB82* and OE‐*CsMYB82* leaves. (A) Gene expression analysis in pTRV: *CsMYB82* leaves. (B) Gene expression analysis in OE‐*CsMYB82* leaves. The RT‐qPCR data were presented as means ± SD values with three biological replicates. “ns” means no difference and asterisks indicate statistical significance (*p < 0.05; **p < 0.01).
**Figure S4:** Sequence, element analysis and screening of the minimum inhibitory concentration of AbA in promoter. (A) Sequence of *CsMYB82* promoter in ‘Zhongcha 108’. (B) Part cis‐acting elements of *CsMYB82* promoter. (C) Screening of minimal AbA concentration of Y1HGold[pAbAi‐*ProCsMYB82*], Y1HGold[pAbAi‐*ProCsCAD4*] bait strain. p53‐pAbAi was used as a control.
**Figure S5:** Subcellular localisation, genetic transformation, identification and functions of *CsMYB1* in tea plant leaves. (A) Subcellular localisation of *CsMYB1* in tobacco leaves. Bar = 50 μm. (B) The OE‐*CsMYB1* and pTRV: *CsMYB1* constructs. (C, D) Confirmation of OE‐*CsMYB1* and pTRV: *CsMYB1* leaves by RT‐qPCR analysis. The RT‐qPCR data were presented as means ± SD values with three biological replicates. “ns” means no difference and asterisks indicate statistical significance (*p < 0.05, **p < 0.01). (E) Disease symptoms on OE‐*CsMYB1* leaves after *C. gloeosporioides* inoculation at 3, 5 days post inoculation (dpi) in ‘Longjing 43’ leaves. Scale bar = 2 cm. (F) The lesion areas of OE‐*CsMYB1* and WT leaves at 5 days post inoculations (dpi). Values are presented as the means ± SD (*n* = 3). Asterisks indicate statistical significance (*p < 0.05). (G) Measurement of the total lignin content in OE‐*CsMYB1* and wild type (WT‐LJ43) leaves after infiltration at 72 h. Values are presented as the means ± SD (*n* = 5). Asterisks indicate statistical significance (****p < 0.0001). (H) Expression analysis of *CsMYB82* by RT‐qPCR on OE‐*CsMYB1* leaves. The RT‐qPCR data were presented as means ± SD values with three biological replicates. “ns” means no difference and asterisks indicate statistical significance (**p < 0.01). (I) Lignin accumulation through phloroglucinol staining in OE‐*CsMYB1* leaves.
**Figure S6:** Subcellular localisation, genetic transformation and identification of *CsbHLH48* into tea plant leaves. (A) Subcellular localisation of *CsbHLH48*. (B) Confirmation of OE‐*CsbHLH48* and pTRV: *CsbHLH48* leaves in ‘Zhongcha 108’ by RT‐qPCR analysis. The RT‐qPCR data were presented as means ± SD values with three biological replicates. “ns” means no difference and asterisks indicate statistical significance (**p < 0.01).
**Figure S7:** Structure diagram and sequence of *CsCAD4* promoter, phylogenetic analysis of *CsCAD4*, and genetic identification of *CsCAD4* in tea plant leaves. (A) Promoter sequence of *CsCAD4*. (B) Structure diagram. (C) Phylogenetic analysis of *CsCAD4* with the homologous genes in other species. Os, *Oryza sativa* ; At, *Arabidopsis thaliana* . (D) Confirmation of OE‐*CsCAD4* and pTRV: *CsCAD4* leaves in ‘Zhongcha 108’ and ‘Longjing43’ by RT‐qPCR analysis. The RT‐qPCR data were presented as means ± SD values with three biological replicates. “ns” means no difference, and asterisks indicate statistical significance (**p < 0.01). (E–G) Expression analysis of *CsCAD4* by RT‐qPCR on pTRV: *CsMYB82*, pTRV: *CsbHLH48*, pTRV: *CsMYB1* and OE‐*CsMYB82*, OE‐*CsbHLH48*, OE‐*CsMYB1* leaves. The RT‐qPCR data were presented as means ± SD values with three biological replicates. “ns” means no difference, and asterisks indicate statistical significance (**p < 0.01).
**Figure S8:** Cultivar‐dependent transcriptional responses and differential lignin accumulation in six tea cultivars after *C. gloeosporioides* infection. (A) Expression analysis of *CsMYB1*, *CsMYB82*, *CsbHLH48*, and *CsCAD4* by RT‐qPCR at 2, 3, and 5 day‐post inoculations (dpi) after *C. gloeosporioides* inoculation. Three independent replicates were conducted for each RT‐qPCR analysis. Different letters above the bars denote significant differences using a Tukey's multiple comparison test followed by two‐way ANOVA (*p* < 0.05). Measurements were performed at the indicated time points, and comparisons were conducted within each time point. (B) Measurement of the total lignin content in six tea cultivar leaves under natural conditions. Values are presented as the means ± SD (*n* = 3). Different letters above the bars denote significant differences using a Tukey's multiple comparison test followed by two‐way ANOVA (*p* < 0.05). Measurements were performed at the indicated time points, and comparisons were conducted within each time point.

**Table S1:** Specific primers used for vector construction. F, forward; R, reverse.

**Table S2:** Specific primers used for RT‐qPCR. F, forward; R, reverse.

**Table S3:** Information about upstream regulatory genes of CsMYB82 screening by yeast one‐hybrid.

**Table S4:** Information about interacting proteins of CsMYB82 screening by yeast two‐hybrid.

**Table S5:** Statistics of DAP‐seq alignment.

**Table S6:** Binding peaks of CsMYB82 by using DNA affinity purification followed by sequencing (DAP‐seq) analysis.

## Data Availability

The data that supports the findings of this study are available in the Supporting Information of this article.
