# Supplementary material for: Regulatory Mechanism of CsMYB1‐CsMYB82/CsbHLH48‑CsCAD4 Model for Resistance Against Colletotrichum gloeosporioides in Camellia sinensis
Source: Plant Biotechnol J. 2026 Apr 3;24(8):4725–47. doi: 10.1111/pbi.70659 (PMC13387894; doi:10.1111/pbi.70659)
Supplement: Supplementary file 1 — Figure S1: Bioinformatic analyses of CsMYB82. (A) Chromosome location of CsMYB82. CsMYB82 is located on chromosome 6 with two SANTs domain. (B) Protein sequence alignment of conserved domain of CsMYB82. At, Arabidopsis thaliana ; Nt, Nicotiana tabacum ; Vv, Vitis vinifera . Figure S2: CsMYB82 phylogenetic analysis, transcriptional activation activity and identification of CsMYB82 transgenic leaves. (A) Phylogenetic analysis of CsMYB82 with the homologous genes in other species. (B) CsMYB82 transactivation assay in yeast. Co‐transformation of AD‐T with BD‐p53 or BD‐Lam into yeast cells was used as positive (Po) or negative controls (Ne), respectively. SD − Trp/X, SD − Trp/X‐α‐Gal; SD − Trp/X/A, SD − Trp/X‐α‐Gal/aureobasidin A. (C) The OE‐CsMYB82 and pTRV: CsMYB82 constructs. (D) Quantitative analysis of CsMYB82 overexpression lines (L1, L2, L3, L4, L5, L6, L7, L8) and wild type (WT). The RT‐qPCR data were presented as means ± SD values with three biological replicates. Asterisks indicate statistical significance (**p < 0.01). (E) Petiole injection. The second leaf position was selected for the experiment. (F, G) Confirmation of Virus‐induced gene silencing (VIGS) and OE‐CsMYB82 leaves by RT‐qPCR analysis. #1, #2, #3, #4, #5 and #6 were referred to the distinct pTRV: CsMYB82 leaves in ‘Longjing 43’. WT(Wild‐type) and pTRV2 as controls. OE#1—OE#11 were referred to the distinct OE‐CsMYB82 leaves in ‘Zhongcha 108’. WT(Wild‐type) and empty vector (EV) as controls. The RT‐qPCR data were presented as means ± SD values with three biological replicates. “ns” means no difference and asterisks indicate statistical significance (*p < 0.05, **p < 0.01). (H) Lignin accumulation through phloroglucinol staining in OE‐CsMYB82 leaves. Scale bar = 100 μm. Figure S3: Gene expression analysis in lignin synthesis pathway in pTRV: CsMYB82 and OE‐CsMYB82 leaves. (A) Gene expression analysis in pTRV: CsMYB82 leaves. (B) Gene expression analysis in OE‐CsMYB82 leaves. The RT‐qPCR data were pre [file PBI-24-4725-s003.zip › pbi70659-sup-0001-FigureS1-S8.docx]

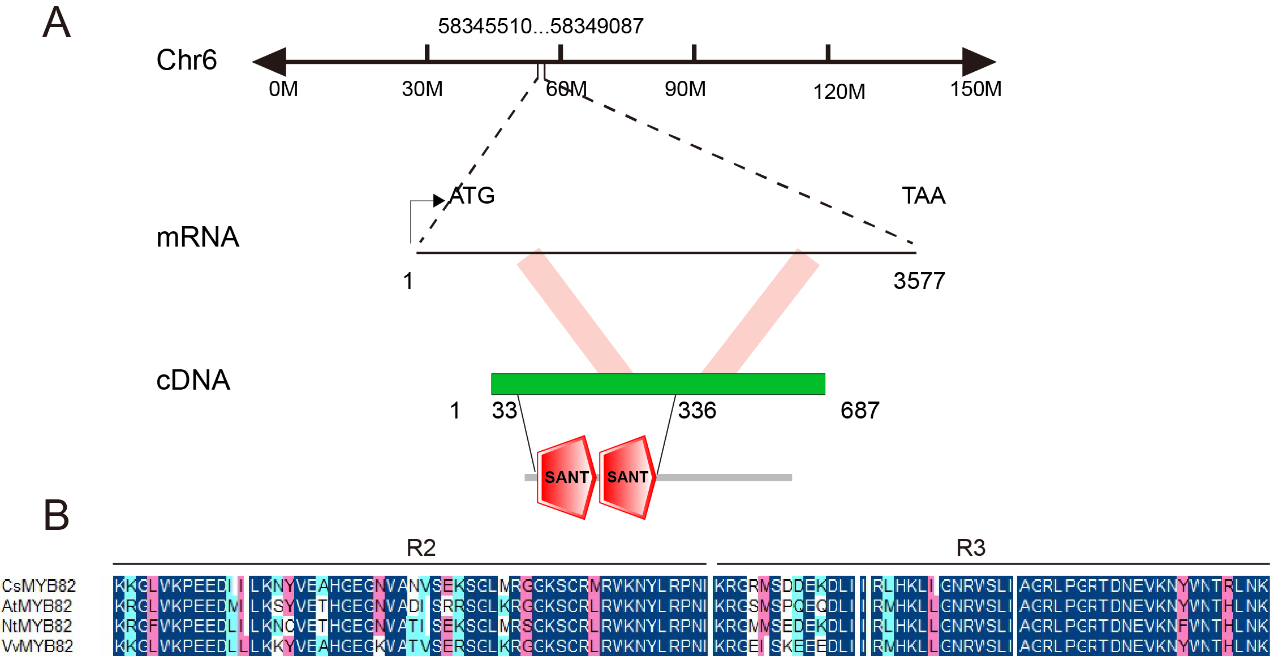


**Figure S1** Bioinformatic analyses of CsMYB82. **A** Chromosome location of *CsMYB82*. *CsMYB82* is located on chromosome 6 with two SANTs domain. **B** Protein sequence alignment of conserved domain of CsMYB82. At, *Arabidopsis thaliana*; Nt, *Nicotiana tabacum*; Vv, *Vitis vinifera*.


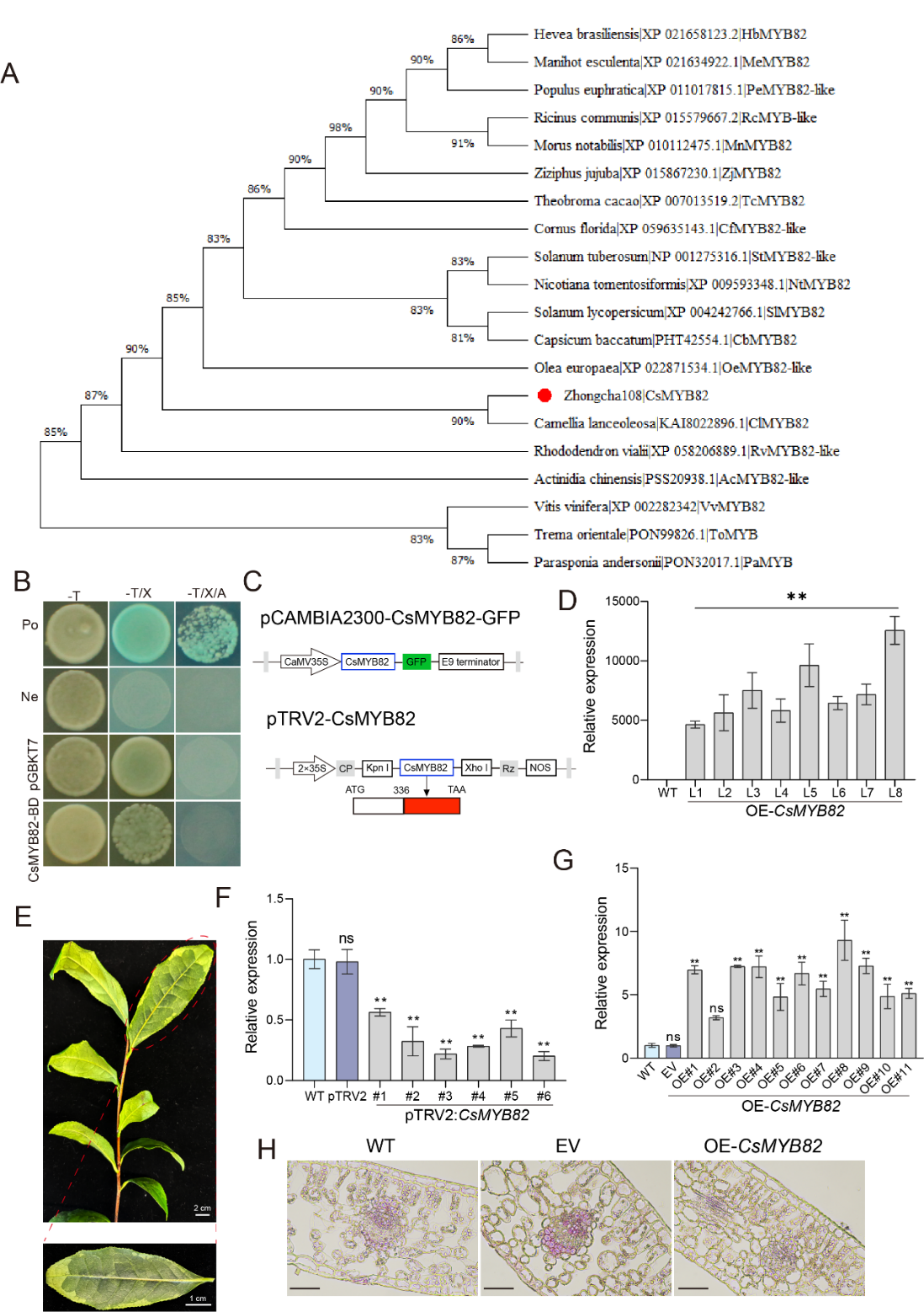


**Figure S2 CsMYB82 phylogenetic analysis, transcriptional activation activity and identification of CsMYB82 transgenic leaves.** **A** Phylogenetic analysis of CsMYB82 with the homologous genes in other species. **B** CsMYB82 transactivation assay in yeast. Co-transformation of AD-T with BD-p53 or BD-Lam into yeast cells was used as positive (Po) or negative controls (Ne), respectively. Abbreviations: SD−Trp/X, SD−Trp/X-α-Gal; SD−Trp/X/A, SD−Trp/X-α-Gal/aureobasidin A. **C** The OE-*CsMYB82* and pTRV: *CsMYB82* constructs. **D** Quantitative analysis of *CsMYB82* overexpression lines (L1、L2、L3、L4、L5、L6、L7、L8) and wild type (WT). The RT-qPCR data were presented as means ± SD values with three biological replicates. Asterisks indicate statistical significance (***P* < 0.01). **E** Petiole injection. The second leaf position was selected for the experiment. **F-G** Confirmation of Virus-induced gene silencing (VIGS) and OE-*CsMYB82* leaves by RT-qPCR analysis. #1, #2, #3, #4, #5 and #6 were referred to the distinct pTRV: *CsMYB82* leaves in ‘Longjing 43’. WT(Wild-type) and pTRV2 as controls. OE#1 - OE#11 were referred to the distinct OE-*CsMYB82* leaves in ‘Zhongcha 108’. WT(Wild-type) and empty vector (EV) as controls. The RT-qPCR data were presented as means ± SD values with three biological replicates. “ns” means no difference and asterisks indicate statistical significance (**P* < 0.05, ***P* < 0.01). **H** Lignin accumulation through phloroglucinol staining in OE-*CsMYB82* leaves. Scale bar = 100 μm.


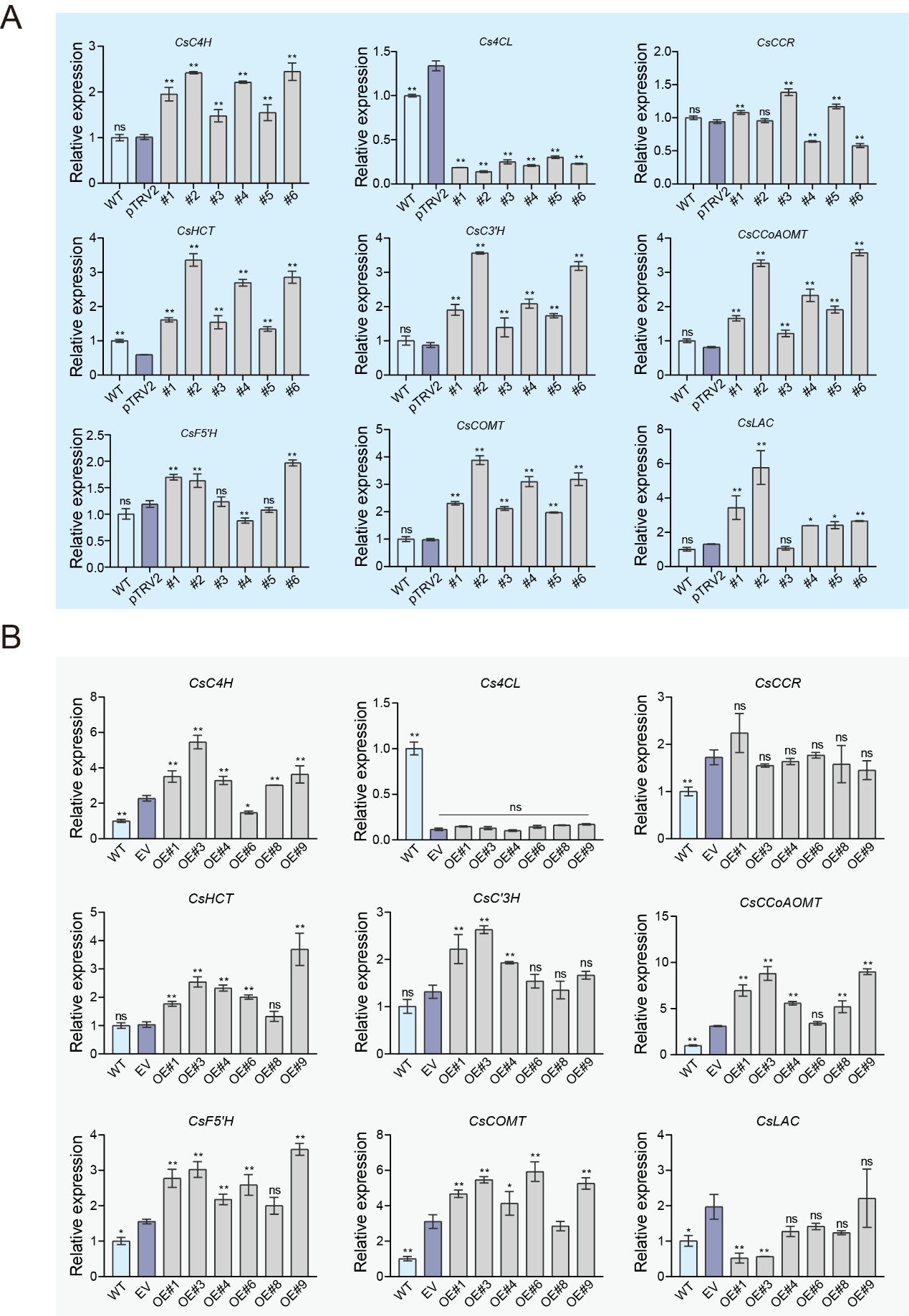


**Figure S3 Gene expression analysis in lignin synthesis pathway in pTRV: *CsMYB82* and OE-*CsMYB82* leaves. A** Gene expression analysis in pTRV: *CsMYB82* leaves. **B** Gene expression analysis in OE-*CsMYB82* leaves. The RT-qPCR data were presented as means ± SD values with three biological replicates. “ns” means no difference and asterisks indicate statistical significance (**P* < 0.05; ***P* < 0.01).


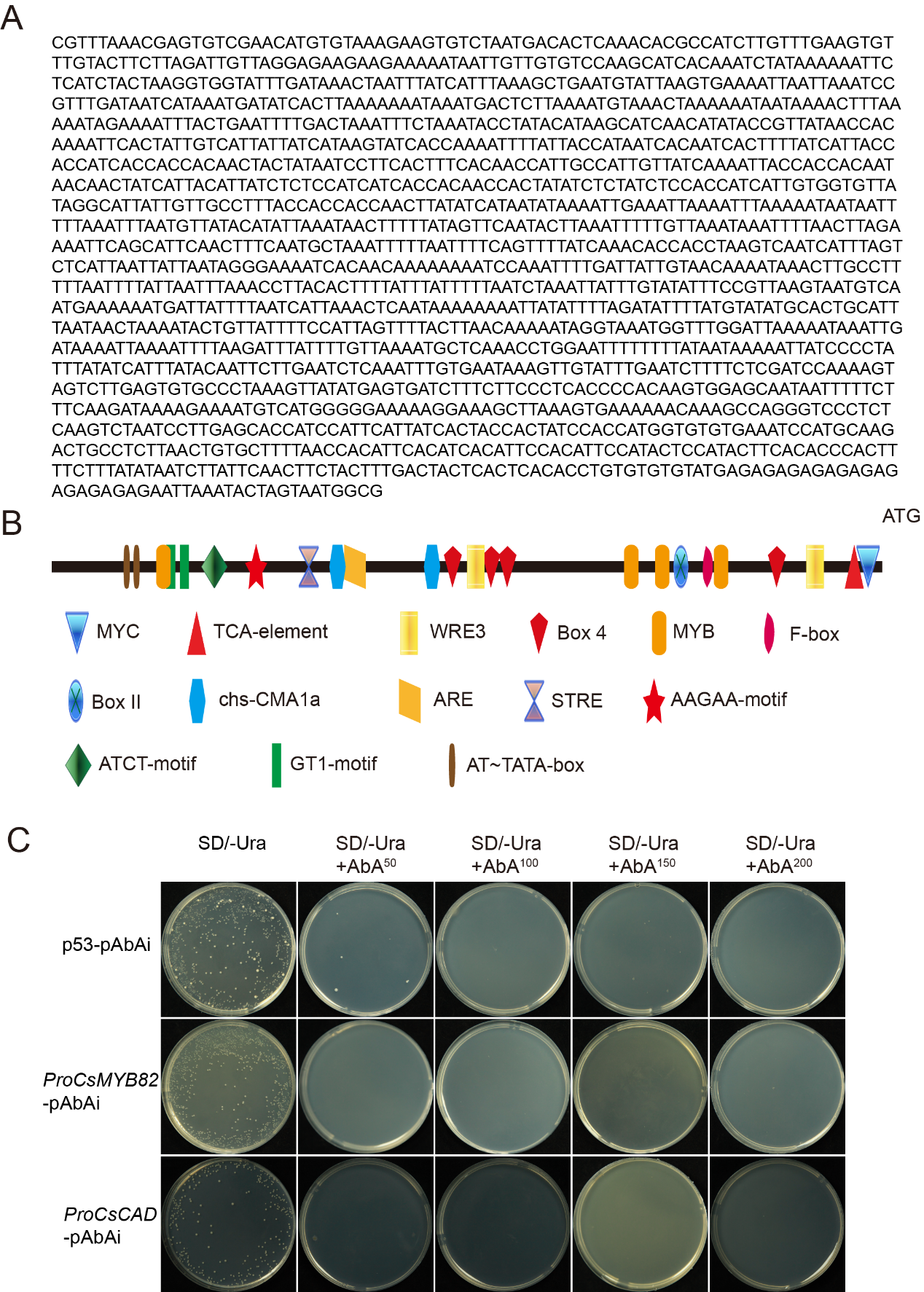


**Figure S4 Sequence, element analysis and screening of the minimum inhibitory concentration of AbA in promoter. A** Sequence of *CsMYB82* promoter in ‘Zhongcha 108’. **B** Part cis-acting elements of *CsMYB82* promoter. **C** Screening of minimal AbA concentration of Y1HGold[pAbAi-*ProCsMYB82*], Y1HGold[pAbAi-*ProCsCAD4*] bait strain. p53-pAbAi was used as a control.


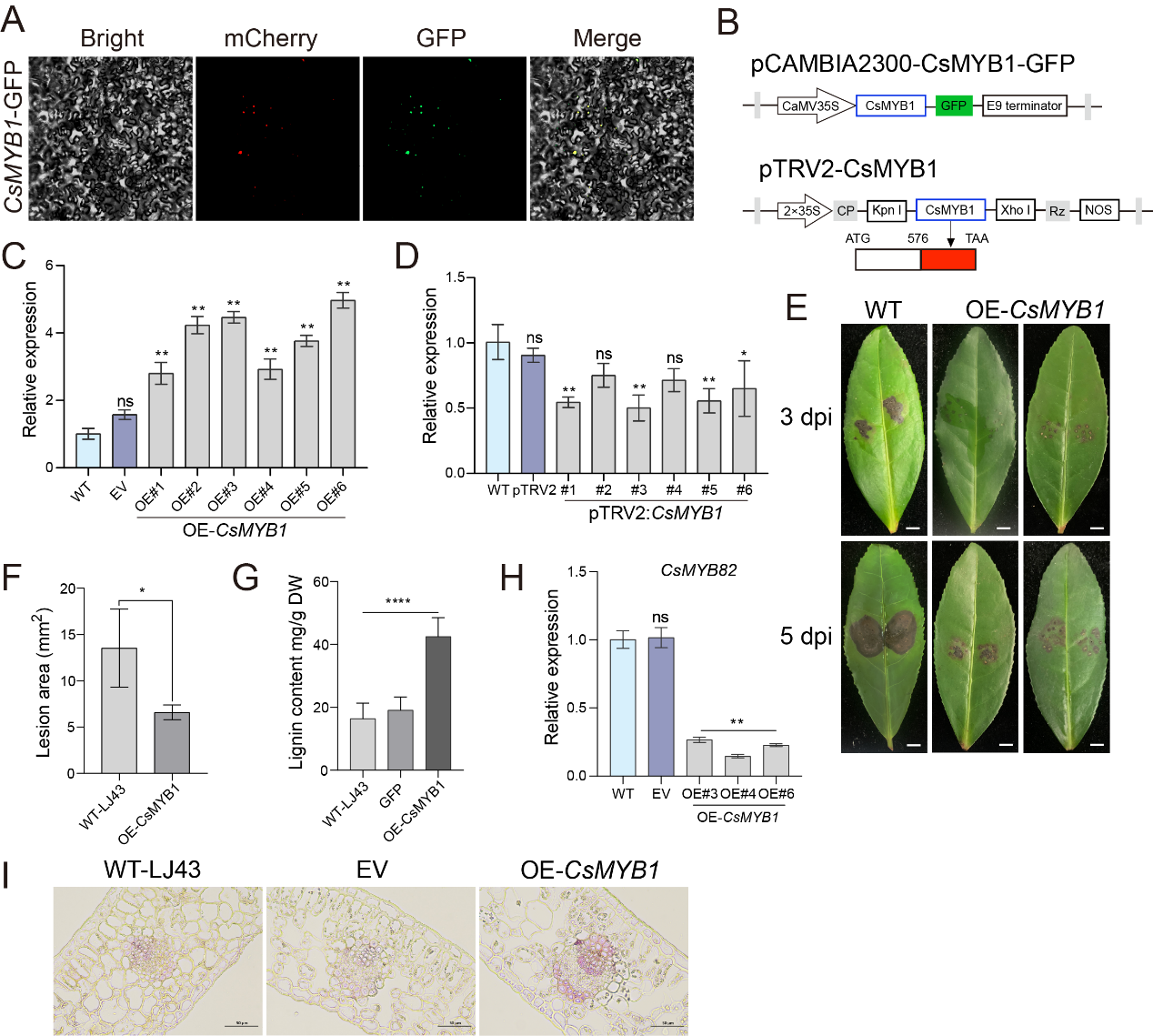


**Figure S5 Subcellular localization, genetic transformation, identification and functions of *CsMYB1* into tea plant leaves.** **A** Subcellular localization of *CsMYB1* in tobacco leaves. Bar = 50 μm. **B** The OE-*CsMYB1* and pTRV: *CsMYB1* constructs. **C-D** Confirmation of OE-*CsMYB1* and pTRV: *CsMYB1* leaves by RT-qPCR analysis. The RT-qPCR data were presented as means ± SD values with three biological replicates. “ns” means no difference and asterisks indicate statistical significance (**P* < 0.05, ***P* < 0.01). **E** Disease symptoms on OE-*CsMYB1* leaves after *C. gloeosporioides* inoculation at 3, 5 days post inoculation (dpi) in ‘Longjing 43’ leaves. Scale bar = 2 cm. **F** The lesion areas of OE-*CsMYB1* and WT leaves at 5 days post inoculations (dpi). Values are presented as the means ± SD (n = 3). Asterisks indicate statistical significance (**P* < 0.05). **G** Measurement of the total lignin content in OE-*CsMYB1* and wild type (WT-LJ43) leaves after infiltrated leaves at 72 hours. Values are presented as the means ± SD (n = 5). Asterisks indicate statistical significance (*****P* < 0.0001). **H** Expression analysis of *CsMYB82* by RT-qPCR on OE-*CsMYB1* leaves. The RT-qPCR data were presented as means ± SD values with three biological replicates. “ns” means no difference and asterisks indicate statistical significance (***P* < 0.01). **I** Lignin accumulation through phloroglucinol staining in OE-*CsMYB1* leaves.


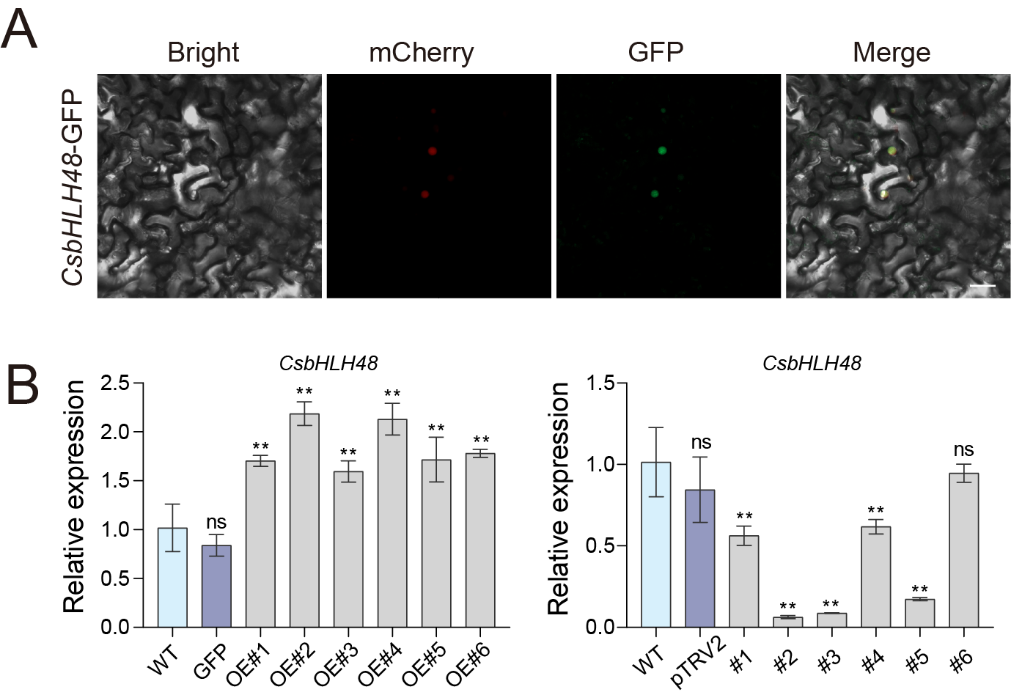


**Figure S6 Subcellular localization, genetic transformation and identification of *CsbHLH48* into tea plant leaves. A** Subcellular localization of *CsbHLH48*. **B** Confirmation of OE-*CsbHLH48* and pTRV: *CsbHLH48* leaves in ‘Zhongcha 108’ by RT-qPCR analysis. The RT-qPCR data were presented as means ± SD values with three biological replicates. “ns” means no difference and asterisks indicate statistical significance (***P* < 0.01).


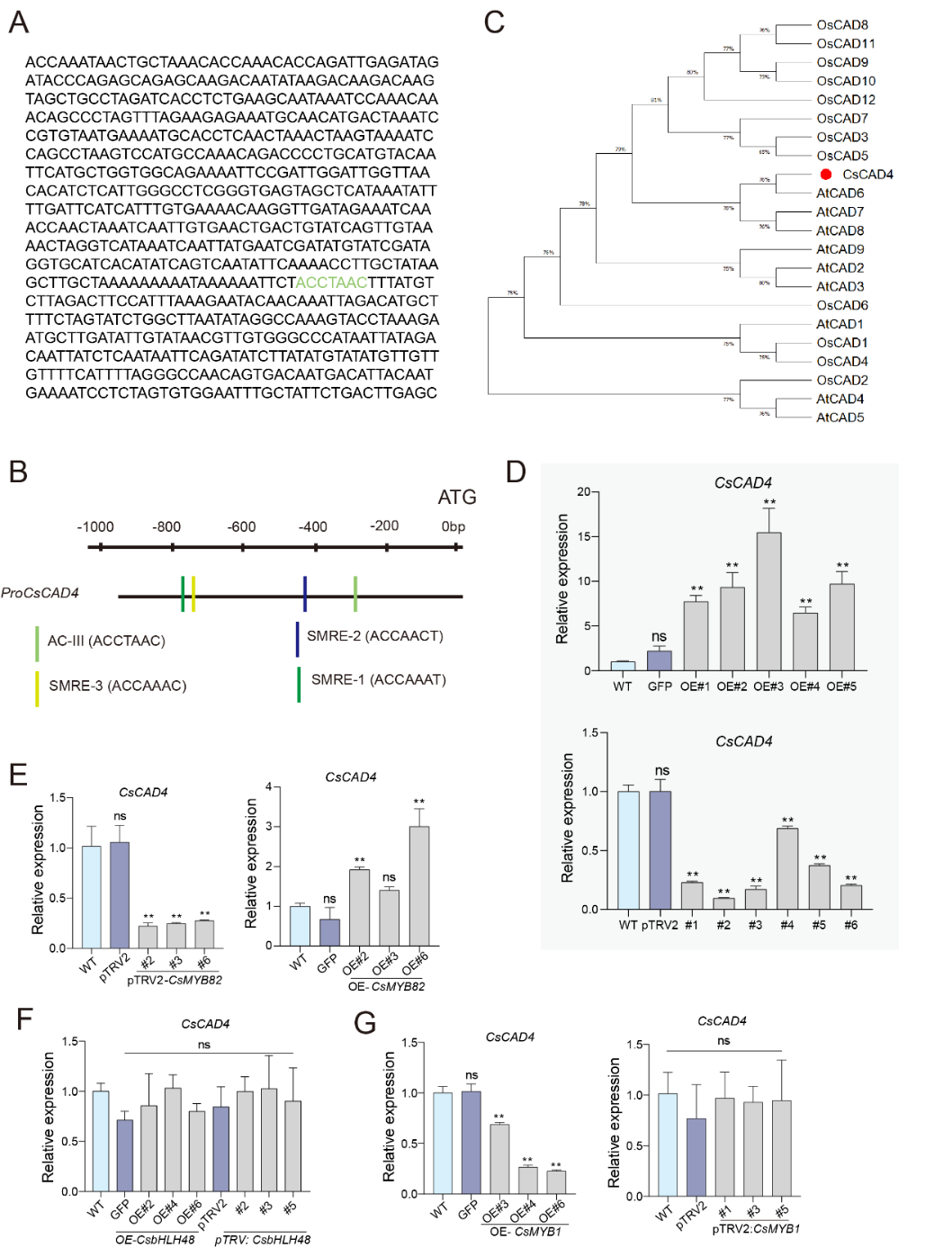


**Figure S7 Structure diagram and sequence of *CsCAD4* promoter, phylogenetic analysis of *CsCAD4*, genetic identification of *CsCAD4* into tea plant leaves.** **A** Promoter sequence of *CsCAD4*. **B** Structure diagram. **C** Phylogenetic analysis of *CsCAD4* with the homologous genes in other species. Os, *Oryza sativa*; At, *Arabidopsis thaliana.* **D** Confirmation of OE-*CsCAD4* and pTRV: *CsCAD4* leaves in ‘Zhongcha 108’ and ‘Longjing43’ by RT-qPCR analysis. The RT-qPCR data were presented as means ± SD values with three biological replicates. “ns” means no difference and asterisks indicate statistical significance (***P* < 0.01). **E-G** Expression analysis of *CsCAD4* by RT-qPCR on pTRV: *CsMYB82*, pTRV: *CsbHLH48*, pTRV: *CsMYB1* and OE-*CsMYB82*, OE-*CsbHLH48*, OE-*CsMYB1* leaves. The RT-qPCR data were presented as means ± SD values with three biological replicates. “ns” means no difference and asterisks indicate statistical significance (***P* < 0.01).


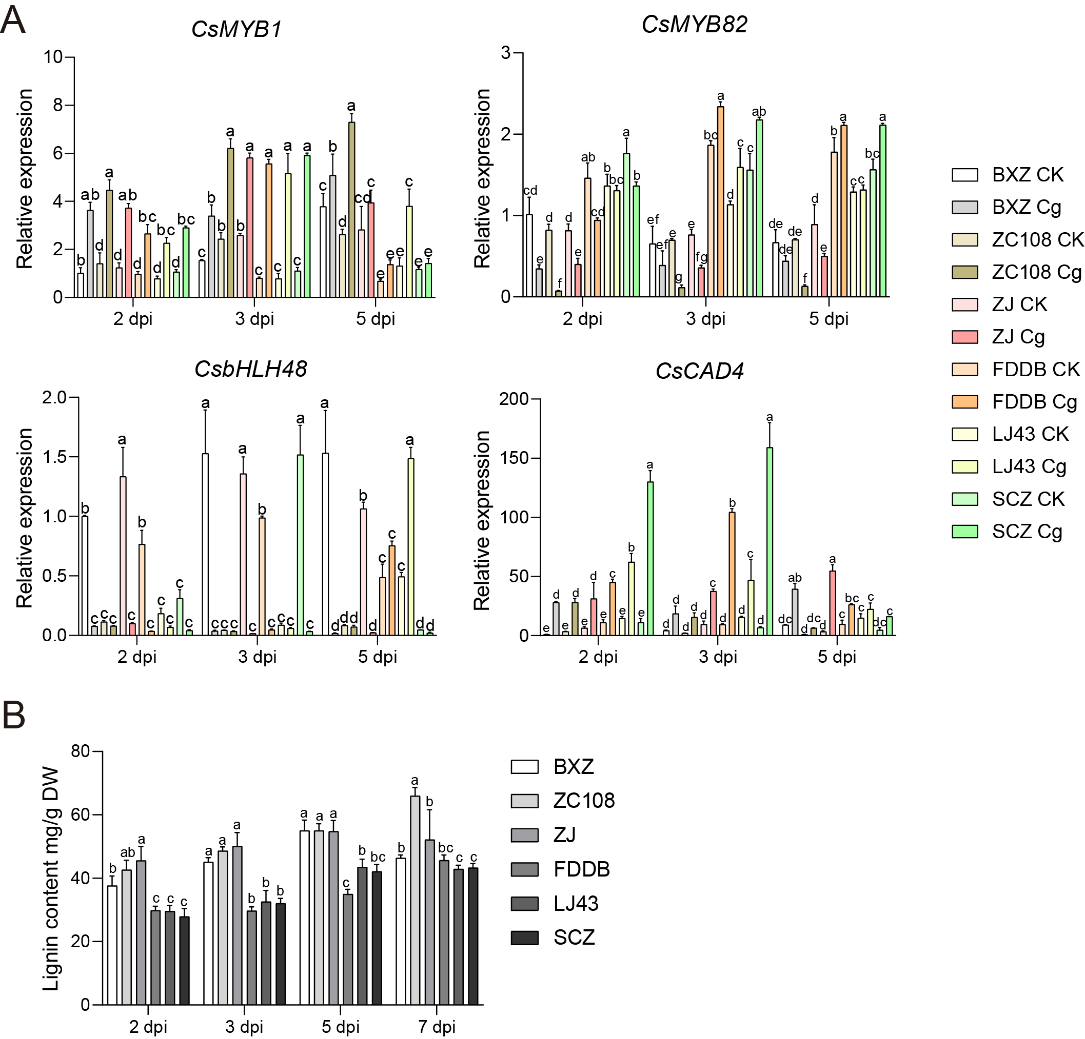


**Figure S8** Cultivar-dependent transcriptional responses and differential lignin accumulation in six tea cultivars after *C. gloeosporioides* infection. **A** Expression analysis of *CsMYB1*, *CsMYB82*, *CsbHLH48*, *CsCAD4* by RT-qPCR at 2, 3 and 5 day-post inoculations (dpi) after *C. gloeosporioides* inoculation. Three independent replicates were conducted for each RT-qPCR analysis. Different letters above the bars denote significant differences using a Tukey’s multiple comparison test followed by two-way ANOVA (*P*<0.05). Measurements were performed at the indicated time points, and comparisons were conducted within each time point. **B** Measurement of the total lignin content in six tea cultivars leaves under natural conditions. Values are presented as the means ± SD (n = 3). Different letters above the bars denote significant differences using a Tukey’s multiple comparison test followed by two-way ANOVA (*P*<0.05). Measurements were performed at the indicated time points, and comparisons were conducted within each time point.
