# Supplementary figures and images for: Regulatory Mechanism of CsMYB1‐CsMYB82/CsbHLH48‑CsCAD4 Model for Resistance Against Colletotrichum gloeosporioides in Camellia sinensis
Source: Plant Biotechnol J. 2026 Apr 3;24(8):4725–47. doi: 10.1111/pbi.70659 (PMC13387894; doi:10.1111/pbi.70659)

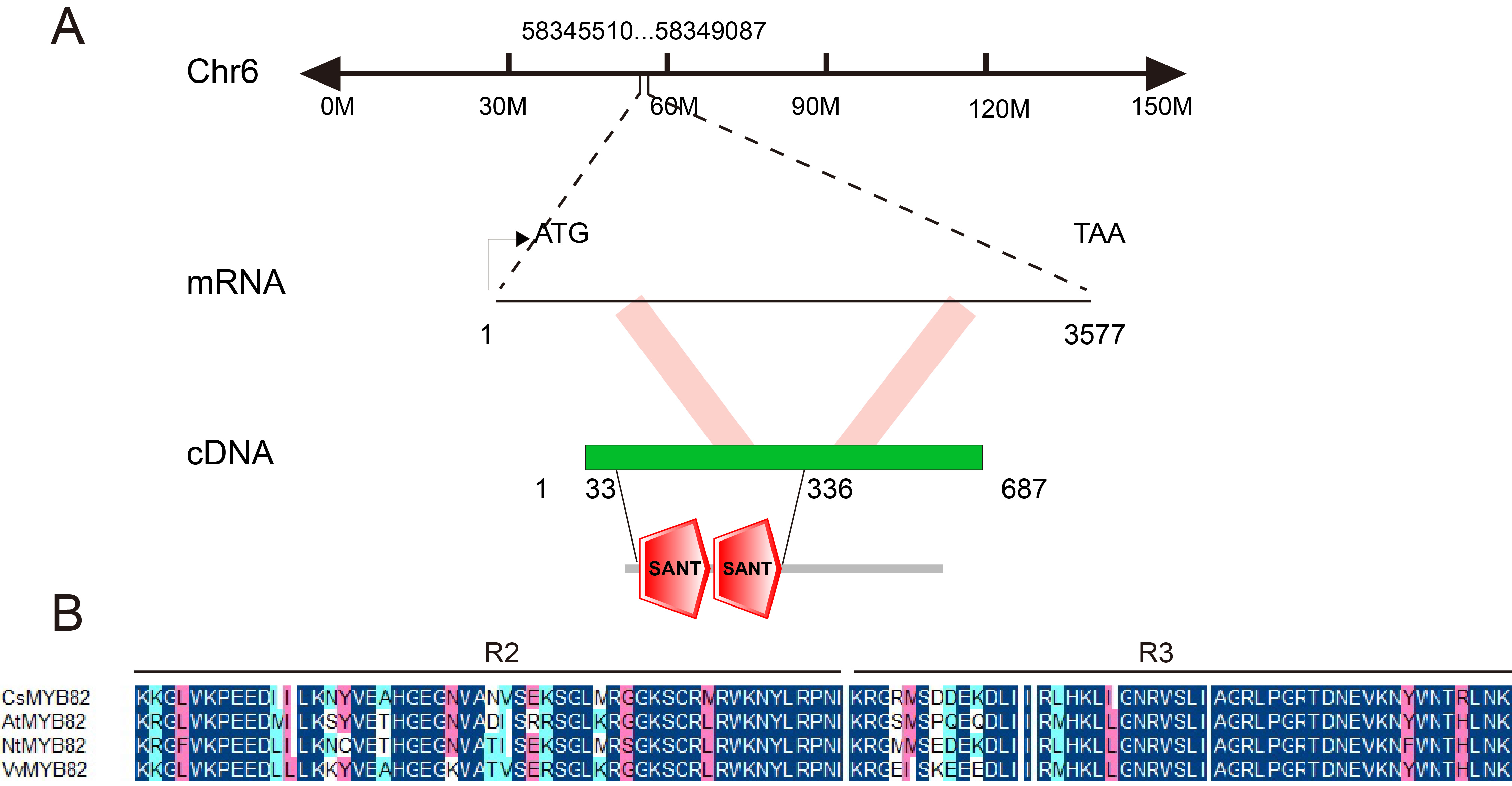

Supplement: Supplementary file 1 — Figure S1: Bioinformatic analyses of CsMYB82. (A) Chromosome location of CsMYB82. CsMYB82 is located on chromosome 6 with two SANTs domain. (B) Protein sequence alignment of conserved domain of CsMYB82. At, Arabidopsis thaliana ; Nt, Nicotiana tabacum ; Vv, Vitis vinifera . Figure S2: CsMYB82 phylogenetic analysis, transcriptional activation activity and identification of CsMYB82 transgenic leaves. (A) Phylogenetic analysis of CsMYB82 with the homologous genes in other species. (B) CsMYB82 transactivation assay in yeast. Co‐transformation of AD‐T with BD‐p53 or BD‐Lam into yeast cells was used as positive (Po) or negative controls (Ne), respectively. SD − Trp/X, SD − Trp/X‐α‐Gal; SD − Trp/X/A, SD − Trp/X‐α‐Gal/aureobasidin A. (C) The OE‐CsMYB82 and pTRV: CsMYB82 constructs. (D) Quantitative analysis of CsMYB82 overexpression lines (L1, L2, L3, L4, L5, L6, L7, L8) and wild type (WT). The RT‐qPCR data were presented as means ± SD values with three biological replicates. Asterisks indicate statistical significance (**p < 0.01). (E) Petiole injection. The second leaf position was selected for the experiment. (F, G) Confirmation of Virus‐induced gene silencing (VIGS) and OE‐CsMYB82 leaves by RT‐qPCR analysis. #1, #2, #3, #4, #5 and #6 were referred to the distinct pTRV: CsMYB82 leaves in ‘Longjing 43’. WT(Wild‐type) and pTRV2 as controls. OE#1—OE#11 were referred to the distinct OE‐CsMYB82 leaves in ‘Zhongcha 108’. WT(Wild‐type) and empty vector (EV) as controls. The RT‐qPCR data were presented as means ± SD values with three biological replicates. “ns” means no difference and asterisks indicate statistical significance (*p < 0.05, **p < 0.01). (H) Lignin accumulation through phloroglucinol staining in OE‐CsMYB82 leaves. Scale bar = 100 μm. Figure S3: Gene expression analysis in lignin synthesis pathway in pTRV: CsMYB82 and OE‐CsMYB82 leaves. (A) Gene expression analysis in pTRV: CsMYB82 leaves. (B) Gene expression analysis in OE‐CsMYB82 leaves. The RT‐qPCR data were pre [file PBI-24-4725-s003.zip › 3_FigS1.jpg]

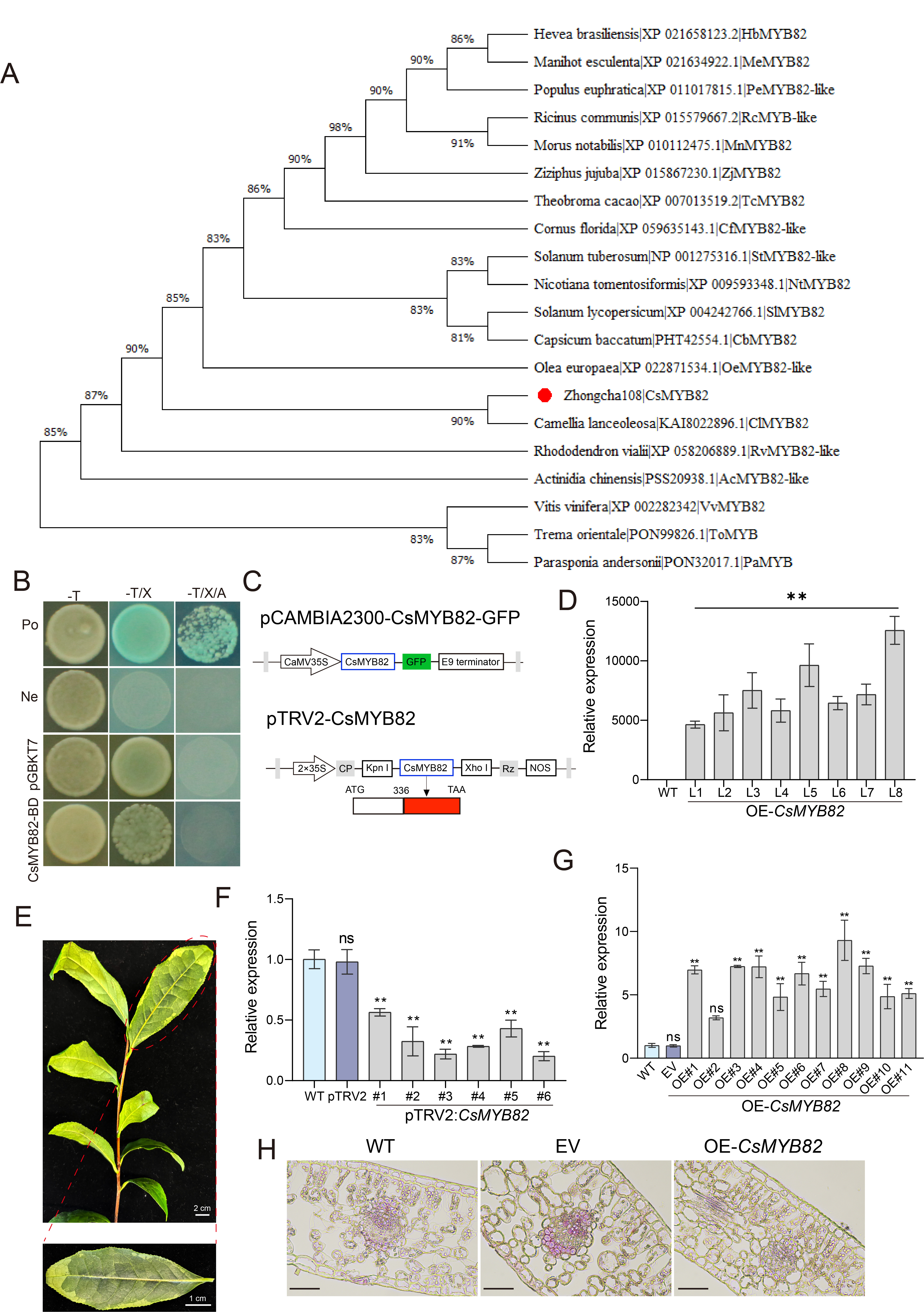

Supplement: Supplementary file 1 — Figure S1: Bioinformatic analyses of CsMYB82. (A) Chromosome location of CsMYB82. CsMYB82 is located on chromosome 6 with two SANTs domain. (B) Protein sequence alignment of conserved domain of CsMYB82. At, Arabidopsis thaliana ; Nt, Nicotiana tabacum ; Vv, Vitis vinifera . Figure S2: CsMYB82 phylogenetic analysis, transcriptional activation activity and identification of CsMYB82 transgenic leaves. (A) Phylogenetic analysis of CsMYB82 with the homologous genes in other species. (B) CsMYB82 transactivation assay in yeast. Co‐transformation of AD‐T with BD‐p53 or BD‐Lam into yeast cells was used as positive (Po) or negative controls (Ne), respectively. SD − Trp/X, SD − Trp/X‐α‐Gal; SD − Trp/X/A, SD − Trp/X‐α‐Gal/aureobasidin A. (C) The OE‐CsMYB82 and pTRV: CsMYB82 constructs. (D) Quantitative analysis of CsMYB82 overexpression lines (L1, L2, L3, L4, L5, L6, L7, L8) and wild type (WT). The RT‐qPCR data were presented as means ± SD values with three biological replicates. Asterisks indicate statistical significance (**p < 0.01). (E) Petiole injection. The second leaf position was selected for the experiment. (F, G) Confirmation of Virus‐induced gene silencing (VIGS) and OE‐CsMYB82 leaves by RT‐qPCR analysis. #1, #2, #3, #4, #5 and #6 were referred to the distinct pTRV: CsMYB82 leaves in ‘Longjing 43’. WT(Wild‐type) and pTRV2 as controls. OE#1—OE#11 were referred to the distinct OE‐CsMYB82 leaves in ‘Zhongcha 108’. WT(Wild‐type) and empty vector (EV) as controls. The RT‐qPCR data were presented as means ± SD values with three biological replicates. “ns” means no difference and asterisks indicate statistical significance (*p < 0.05, **p < 0.01). (H) Lignin accumulation through phloroglucinol staining in OE‐CsMYB82 leaves. Scale bar = 100 μm. Figure S3: Gene expression analysis in lignin synthesis pathway in pTRV: CsMYB82 and OE‐CsMYB82 leaves. (A) Gene expression analysis in pTRV: CsMYB82 leaves. (B) Gene expression analysis in OE‐CsMYB82 leaves. The RT‐qPCR data were pre [file PBI-24-4725-s003.zip › 3_FigS2.jpg]

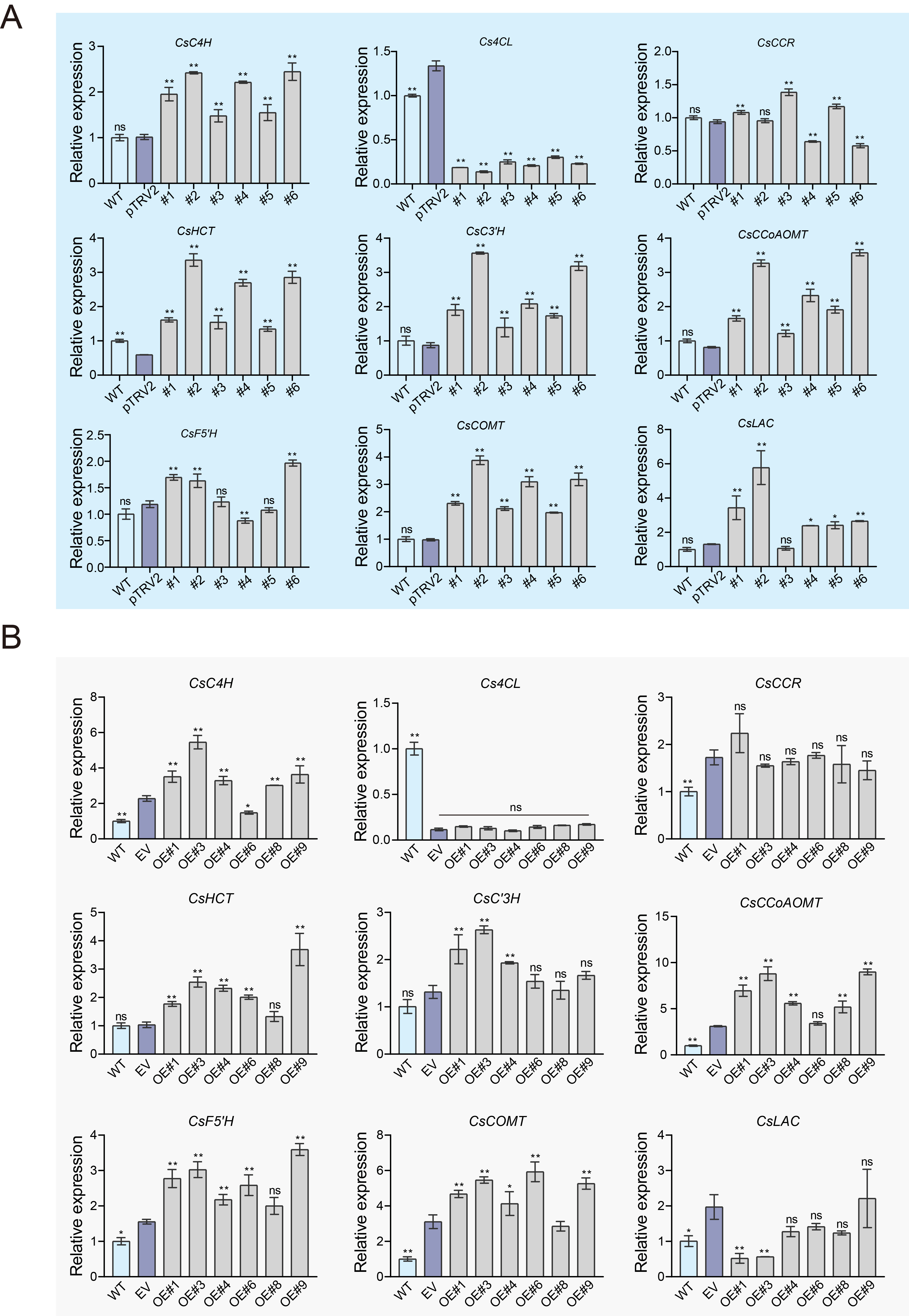

Supplement: Supplementary file 1 — Figure S1: Bioinformatic analyses of CsMYB82. (A) Chromosome location of CsMYB82. CsMYB82 is located on chromosome 6 with two SANTs domain. (B) Protein sequence alignment of conserved domain of CsMYB82. At, Arabidopsis thaliana ; Nt, Nicotiana tabacum ; Vv, Vitis vinifera . Figure S2: CsMYB82 phylogenetic analysis, transcriptional activation activity and identification of CsMYB82 transgenic leaves. (A) Phylogenetic analysis of CsMYB82 with the homologous genes in other species. (B) CsMYB82 transactivation assay in yeast. Co‐transformation of AD‐T with BD‐p53 or BD‐Lam into yeast cells was used as positive (Po) or negative controls (Ne), respectively. SD − Trp/X, SD − Trp/X‐α‐Gal; SD − Trp/X/A, SD − Trp/X‐α‐Gal/aureobasidin A. (C) The OE‐CsMYB82 and pTRV: CsMYB82 constructs. (D) Quantitative analysis of CsMYB82 overexpression lines (L1, L2, L3, L4, L5, L6, L7, L8) and wild type (WT). The RT‐qPCR data were presented as means ± SD values with three biological replicates. Asterisks indicate statistical significance (**p < 0.01). (E) Petiole injection. The second leaf position was selected for the experiment. (F, G) Confirmation of Virus‐induced gene silencing (VIGS) and OE‐CsMYB82 leaves by RT‐qPCR analysis. #1, #2, #3, #4, #5 and #6 were referred to the distinct pTRV: CsMYB82 leaves in ‘Longjing 43’. WT(Wild‐type) and pTRV2 as controls. OE#1—OE#11 were referred to the distinct OE‐CsMYB82 leaves in ‘Zhongcha 108’. WT(Wild‐type) and empty vector (EV) as controls. The RT‐qPCR data were presented as means ± SD values with three biological replicates. “ns” means no difference and asterisks indicate statistical significance (*p < 0.05, **p < 0.01). (H) Lignin accumulation through phloroglucinol staining in OE‐CsMYB82 leaves. Scale bar = 100 μm. Figure S3: Gene expression analysis in lignin synthesis pathway in pTRV: CsMYB82 and OE‐CsMYB82 leaves. (A) Gene expression analysis in pTRV: CsMYB82 leaves. (B) Gene expression analysis in OE‐CsMYB82 leaves. The RT‐qPCR data were pre [file PBI-24-4725-s003.zip › 3_FigS3.jpg]

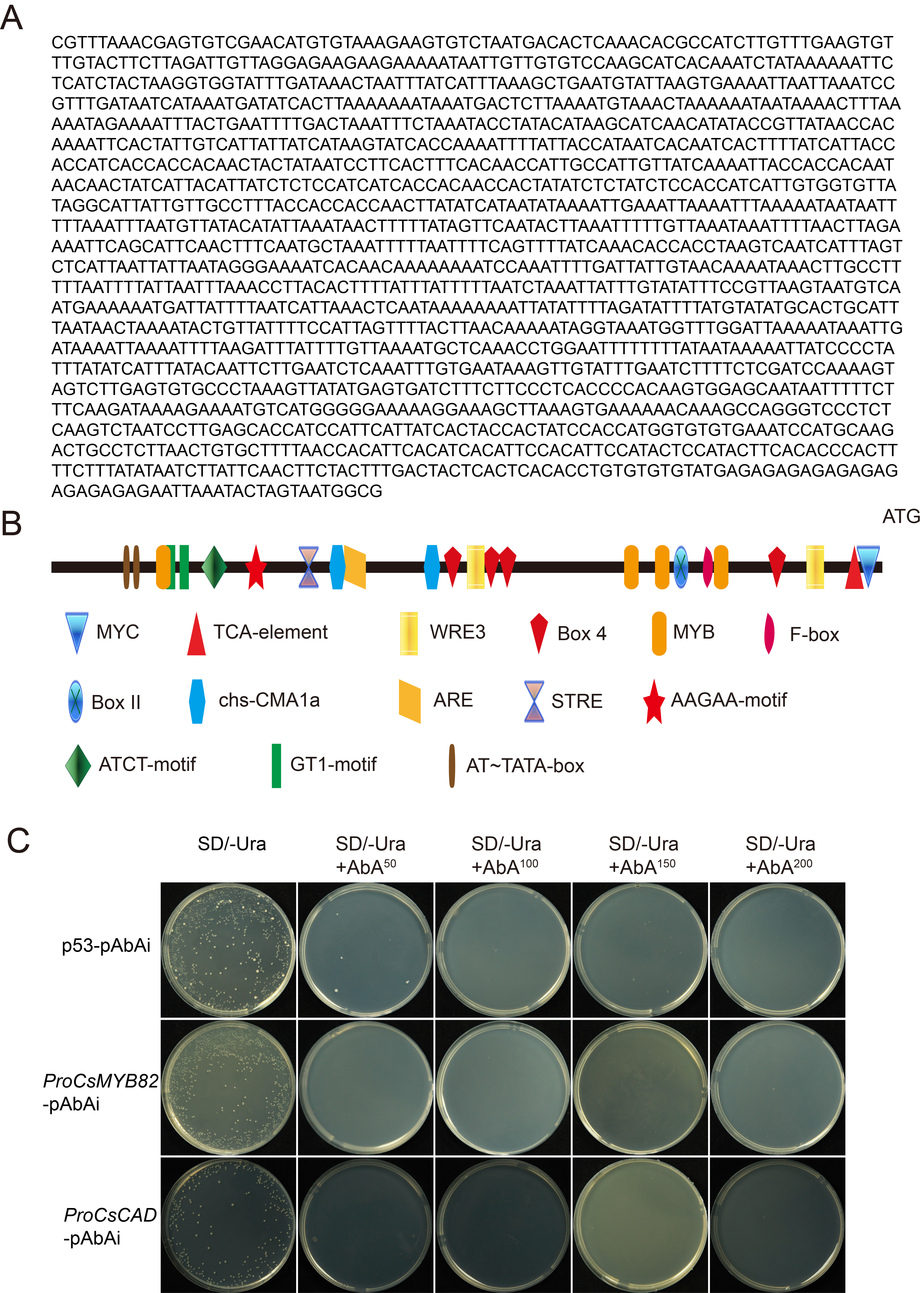

Supplement: Supplementary file 1 — Figure S1: Bioinformatic analyses of CsMYB82. (A) Chromosome location of CsMYB82. CsMYB82 is located on chromosome 6 with two SANTs domain. (B) Protein sequence alignment of conserved domain of CsMYB82. At, Arabidopsis thaliana ; Nt, Nicotiana tabacum ; Vv, Vitis vinifera . Figure S2: CsMYB82 phylogenetic analysis, transcriptional activation activity and identification of CsMYB82 transgenic leaves. (A) Phylogenetic analysis of CsMYB82 with the homologous genes in other species. (B) CsMYB82 transactivation assay in yeast. Co‐transformation of AD‐T with BD‐p53 or BD‐Lam into yeast cells was used as positive (Po) or negative controls (Ne), respectively. SD − Trp/X, SD − Trp/X‐α‐Gal; SD − Trp/X/A, SD − Trp/X‐α‐Gal/aureobasidin A. (C) The OE‐CsMYB82 and pTRV: CsMYB82 constructs. (D) Quantitative analysis of CsMYB82 overexpression lines (L1, L2, L3, L4, L5, L6, L7, L8) and wild type (WT). The RT‐qPCR data were presented as means ± SD values with three biological replicates. Asterisks indicate statistical significance (**p < 0.01). (E) Petiole injection. The second leaf position was selected for the experiment. (F, G) Confirmation of Virus‐induced gene silencing (VIGS) and OE‐CsMYB82 leaves by RT‐qPCR analysis. #1, #2, #3, #4, #5 and #6 were referred to the distinct pTRV: CsMYB82 leaves in ‘Longjing 43’. WT(Wild‐type) and pTRV2 as controls. OE#1—OE#11 were referred to the distinct OE‐CsMYB82 leaves in ‘Zhongcha 108’. WT(Wild‐type) and empty vector (EV) as controls. The RT‐qPCR data were presented as means ± SD values with three biological replicates. “ns” means no difference and asterisks indicate statistical significance (*p < 0.05, **p < 0.01). (H) Lignin accumulation through phloroglucinol staining in OE‐CsMYB82 leaves. Scale bar = 100 μm. Figure S3: Gene expression analysis in lignin synthesis pathway in pTRV: CsMYB82 and OE‐CsMYB82 leaves. (A) Gene expression analysis in pTRV: CsMYB82 leaves. (B) Gene expression analysis in OE‐CsMYB82 leaves. The RT‐qPCR data were pre [file PBI-24-4725-s003.zip › 3_FigS4.jpg]

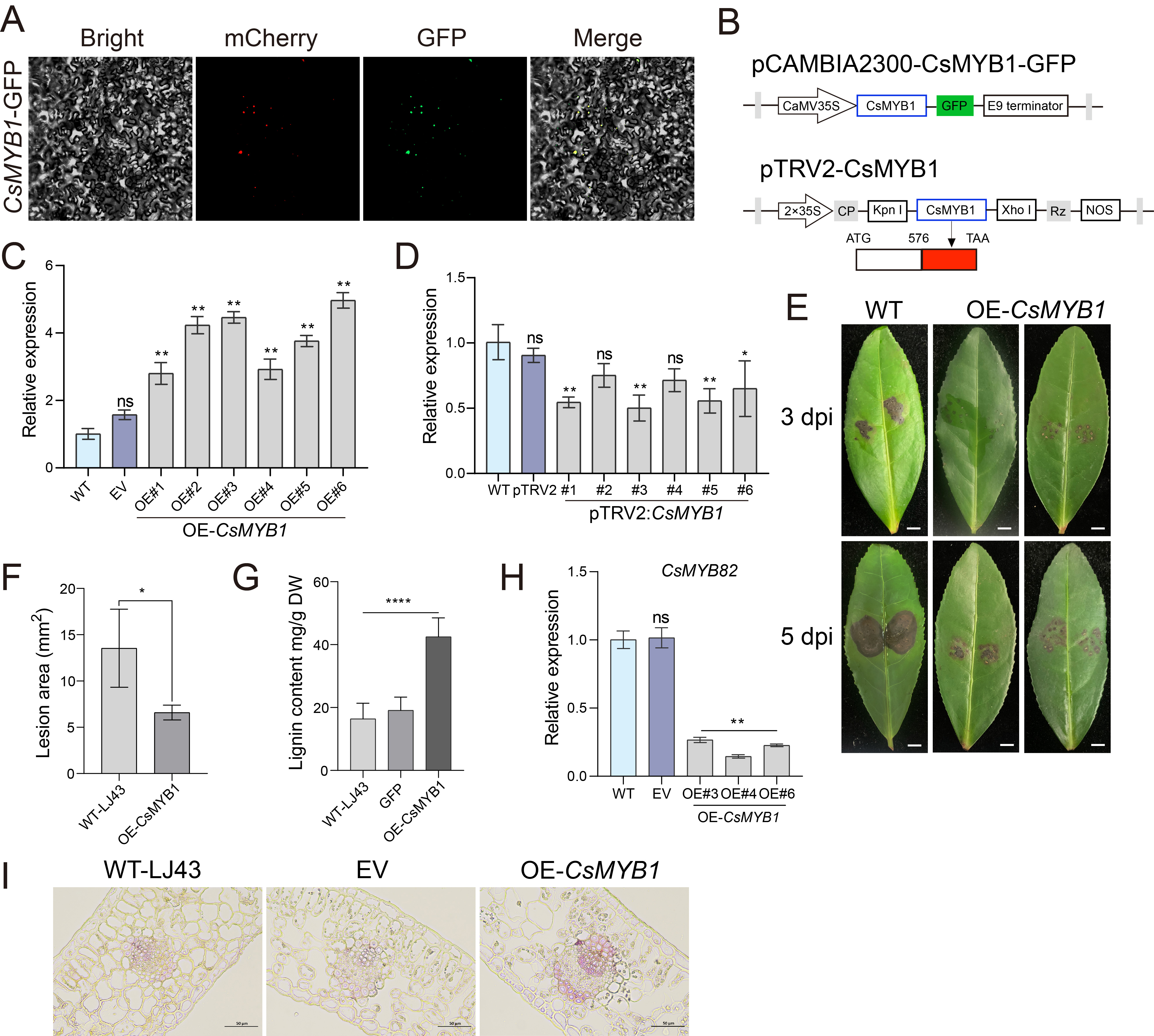

Supplement: Supplementary file 1 — Figure S1: Bioinformatic analyses of CsMYB82. (A) Chromosome location of CsMYB82. CsMYB82 is located on chromosome 6 with two SANTs domain. (B) Protein sequence alignment of conserved domain of CsMYB82. At, Arabidopsis thaliana ; Nt, Nicotiana tabacum ; Vv, Vitis vinifera . Figure S2: CsMYB82 phylogenetic analysis, transcriptional activation activity and identification of CsMYB82 transgenic leaves. (A) Phylogenetic analysis of CsMYB82 with the homologous genes in other species. (B) CsMYB82 transactivation assay in yeast. Co‐transformation of AD‐T with BD‐p53 or BD‐Lam into yeast cells was used as positive (Po) or negative controls (Ne), respectively. SD − Trp/X, SD − Trp/X‐α‐Gal; SD − Trp/X/A, SD − Trp/X‐α‐Gal/aureobasidin A. (C) The OE‐CsMYB82 and pTRV: CsMYB82 constructs. (D) Quantitative analysis of CsMYB82 overexpression lines (L1, L2, L3, L4, L5, L6, L7, L8) and wild type (WT). The RT‐qPCR data were presented as means ± SD values with three biological replicates. Asterisks indicate statistical significance (**p < 0.01). (E) Petiole injection. The second leaf position was selected for the experiment. (F, G) Confirmation of Virus‐induced gene silencing (VIGS) and OE‐CsMYB82 leaves by RT‐qPCR analysis. #1, #2, #3, #4, #5 and #6 were referred to the distinct pTRV: CsMYB82 leaves in ‘Longjing 43’. WT(Wild‐type) and pTRV2 as controls. OE#1—OE#11 were referred to the distinct OE‐CsMYB82 leaves in ‘Zhongcha 108’. WT(Wild‐type) and empty vector (EV) as controls. The RT‐qPCR data were presented as means ± SD values with three biological replicates. “ns” means no difference and asterisks indicate statistical significance (*p < 0.05, **p < 0.01). (H) Lignin accumulation through phloroglucinol staining in OE‐CsMYB82 leaves. Scale bar = 100 μm. Figure S3: Gene expression analysis in lignin synthesis pathway in pTRV: CsMYB82 and OE‐CsMYB82 leaves. (A) Gene expression analysis in pTRV: CsMYB82 leaves. (B) Gene expression analysis in OE‐CsMYB82 leaves. The RT‐qPCR data were pre [file PBI-24-4725-s003.zip › 3_FigS5.jpg]

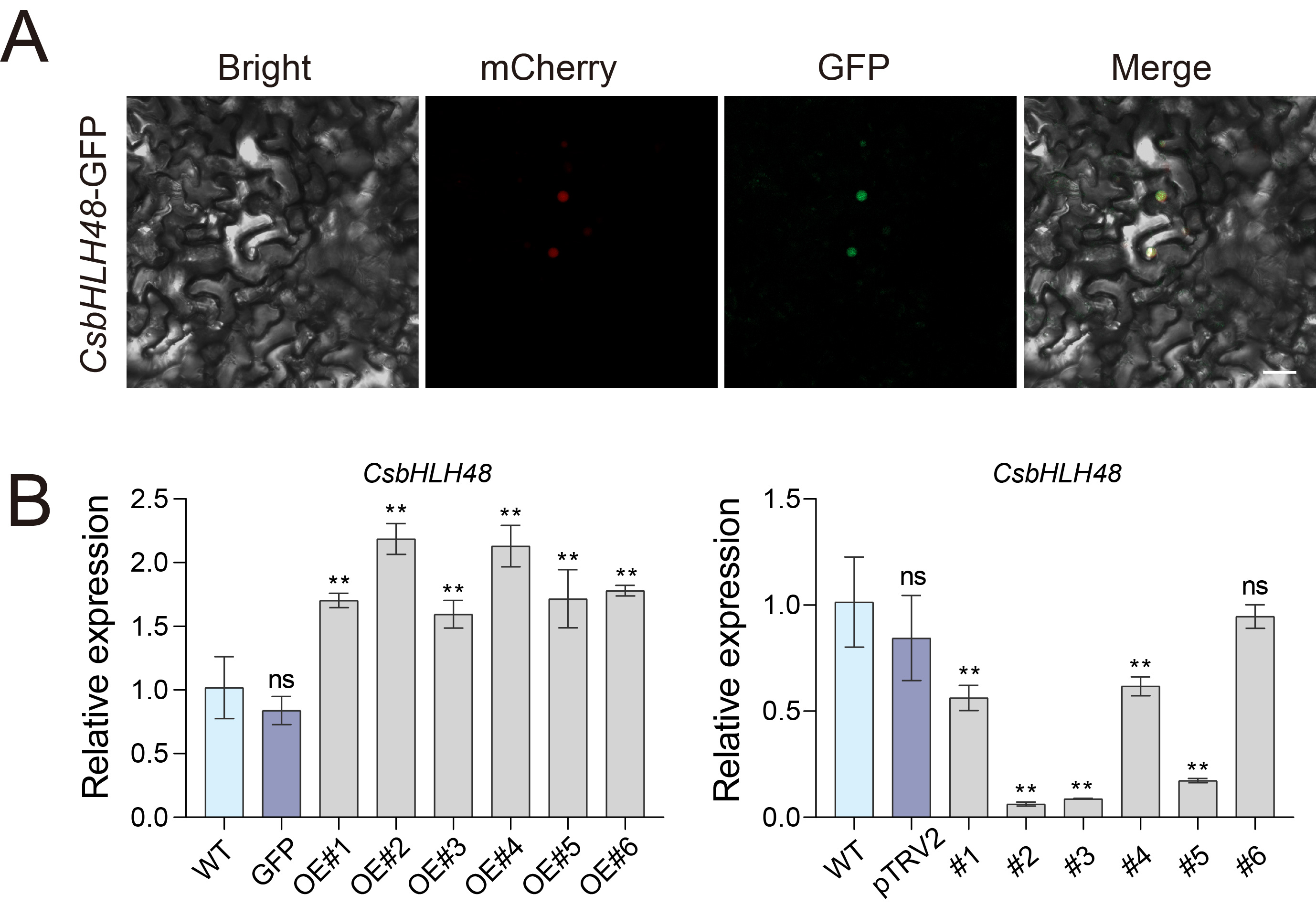

Supplement: Supplementary file 1 — Figure S1: Bioinformatic analyses of CsMYB82. (A) Chromosome location of CsMYB82. CsMYB82 is located on chromosome 6 with two SANTs domain. (B) Protein sequence alignment of conserved domain of CsMYB82. At, Arabidopsis thaliana ; Nt, Nicotiana tabacum ; Vv, Vitis vinifera . Figure S2: CsMYB82 phylogenetic analysis, transcriptional activation activity and identification of CsMYB82 transgenic leaves. (A) Phylogenetic analysis of CsMYB82 with the homologous genes in other species. (B) CsMYB82 transactivation assay in yeast. Co‐transformation of AD‐T with BD‐p53 or BD‐Lam into yeast cells was used as positive (Po) or negative controls (Ne), respectively. SD − Trp/X, SD − Trp/X‐α‐Gal; SD − Trp/X/A, SD − Trp/X‐α‐Gal/aureobasidin A. (C) The OE‐CsMYB82 and pTRV: CsMYB82 constructs. (D) Quantitative analysis of CsMYB82 overexpression lines (L1, L2, L3, L4, L5, L6, L7, L8) and wild type (WT). The RT‐qPCR data were presented as means ± SD values with three biological replicates. Asterisks indicate statistical significance (**p < 0.01). (E) Petiole injection. The second leaf position was selected for the experiment. (F, G) Confirmation of Virus‐induced gene silencing (VIGS) and OE‐CsMYB82 leaves by RT‐qPCR analysis. #1, #2, #3, #4, #5 and #6 were referred to the distinct pTRV: CsMYB82 leaves in ‘Longjing 43’. WT(Wild‐type) and pTRV2 as controls. OE#1—OE#11 were referred to the distinct OE‐CsMYB82 leaves in ‘Zhongcha 108’. WT(Wild‐type) and empty vector (EV) as controls. The RT‐qPCR data were presented as means ± SD values with three biological replicates. “ns” means no difference and asterisks indicate statistical significance (*p < 0.05, **p < 0.01). (H) Lignin accumulation through phloroglucinol staining in OE‐CsMYB82 leaves. Scale bar = 100 μm. Figure S3: Gene expression analysis in lignin synthesis pathway in pTRV: CsMYB82 and OE‐CsMYB82 leaves. (A) Gene expression analysis in pTRV: CsMYB82 leaves. (B) Gene expression analysis in OE‐CsMYB82 leaves. The RT‐qPCR data were pre [file PBI-24-4725-s003.zip › 3_FigS6.jpg]

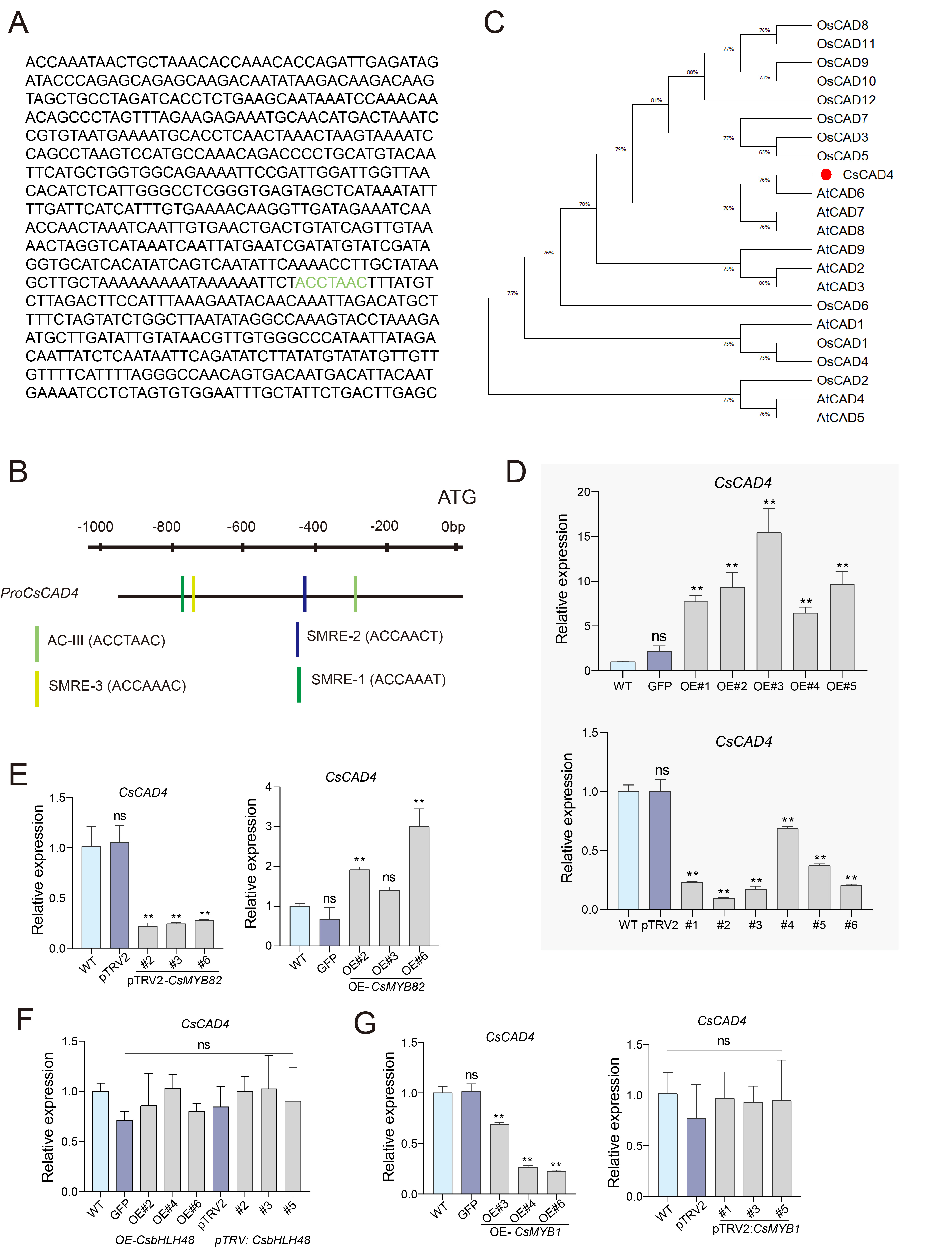

Supplement: Supplementary file 1 — Figure S1: Bioinformatic analyses of CsMYB82. (A) Chromosome location of CsMYB82. CsMYB82 is located on chromosome 6 with two SANTs domain. (B) Protein sequence alignment of conserved domain of CsMYB82. At, Arabidopsis thaliana ; Nt, Nicotiana tabacum ; Vv, Vitis vinifera . Figure S2: CsMYB82 phylogenetic analysis, transcriptional activation activity and identification of CsMYB82 transgenic leaves. (A) Phylogenetic analysis of CsMYB82 with the homologous genes in other species. (B) CsMYB82 transactivation assay in yeast. Co‐transformation of AD‐T with BD‐p53 or BD‐Lam into yeast cells was used as positive (Po) or negative controls (Ne), respectively. SD − Trp/X, SD − Trp/X‐α‐Gal; SD − Trp/X/A, SD − Trp/X‐α‐Gal/aureobasidin A. (C) The OE‐CsMYB82 and pTRV: CsMYB82 constructs. (D) Quantitative analysis of CsMYB82 overexpression lines (L1, L2, L3, L4, L5, L6, L7, L8) and wild type (WT). The RT‐qPCR data were presented as means ± SD values with three biological replicates. Asterisks indicate statistical significance (**p < 0.01). (E) Petiole injection. The second leaf position was selected for the experiment. (F, G) Confirmation of Virus‐induced gene silencing (VIGS) and OE‐CsMYB82 leaves by RT‐qPCR analysis. #1, #2, #3, #4, #5 and #6 were referred to the distinct pTRV: CsMYB82 leaves in ‘Longjing 43’. WT(Wild‐type) and pTRV2 as controls. OE#1—OE#11 were referred to the distinct OE‐CsMYB82 leaves in ‘Zhongcha 108’. WT(Wild‐type) and empty vector (EV) as controls. The RT‐qPCR data were presented as means ± SD values with three biological replicates. “ns” means no difference and asterisks indicate statistical significance (*p < 0.05, **p < 0.01). (H) Lignin accumulation through phloroglucinol staining in OE‐CsMYB82 leaves. Scale bar = 100 μm. Figure S3: Gene expression analysis in lignin synthesis pathway in pTRV: CsMYB82 and OE‐CsMYB82 leaves. (A) Gene expression analysis in pTRV: CsMYB82 leaves. (B) Gene expression analysis in OE‐CsMYB82 leaves. The RT‐qPCR data were pre [file PBI-24-4725-s003.zip › 3_FigS7.jpg]

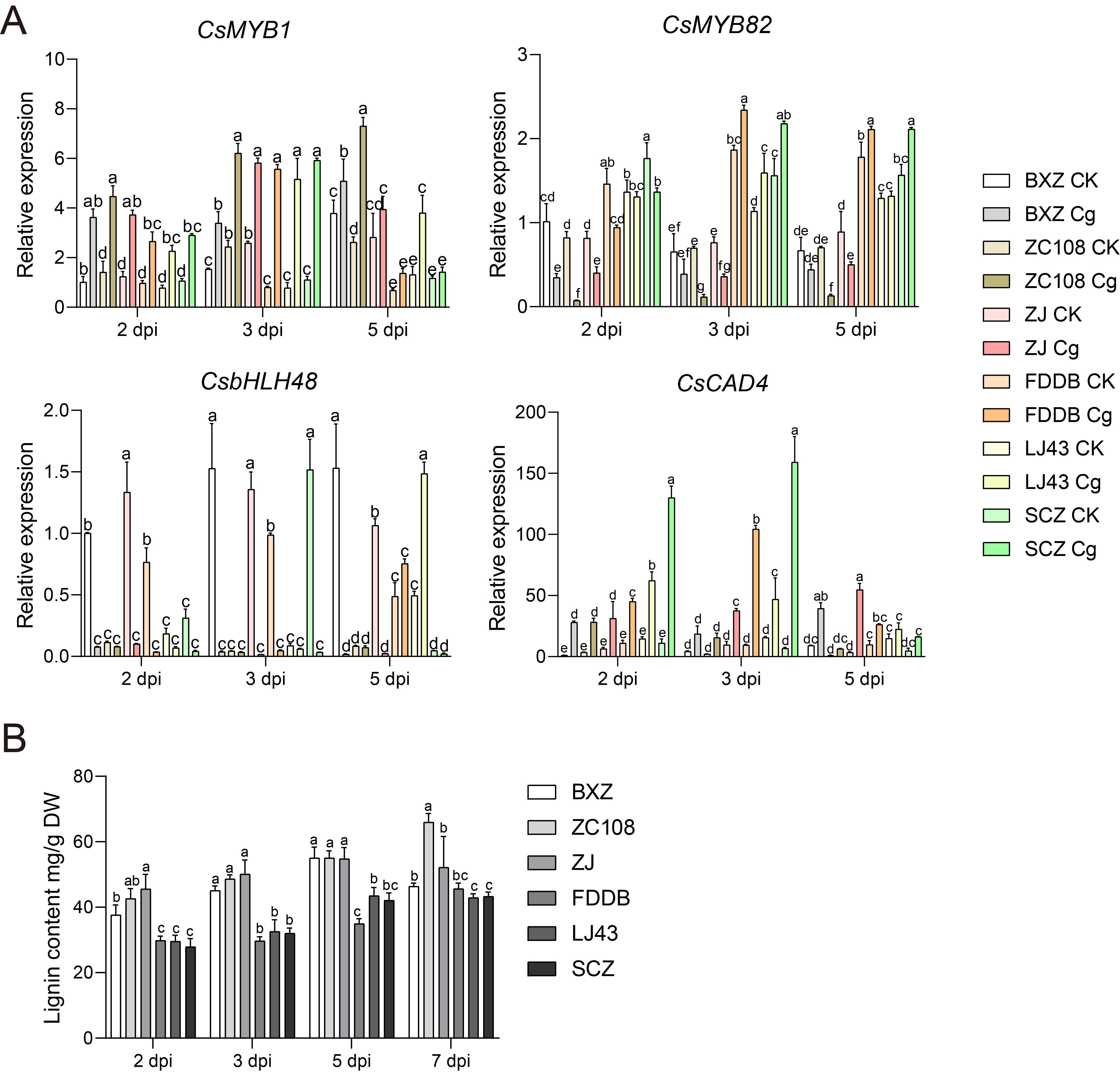

Supplement: Supplementary file 1 — Figure S1: Bioinformatic analyses of CsMYB82. (A) Chromosome location of CsMYB82. CsMYB82 is located on chromosome 6 with two SANTs domain. (B) Protein sequence alignment of conserved domain of CsMYB82. At, Arabidopsis thaliana ; Nt, Nicotiana tabacum ; Vv, Vitis vinifera . Figure S2: CsMYB82 phylogenetic analysis, transcriptional activation activity and identification of CsMYB82 transgenic leaves. (A) Phylogenetic analysis of CsMYB82 with the homologous genes in other species. (B) CsMYB82 transactivation assay in yeast. Co‐transformation of AD‐T with BD‐p53 or BD‐Lam into yeast cells was used as positive (Po) or negative controls (Ne), respectively. SD − Trp/X, SD − Trp/X‐α‐Gal; SD − Trp/X/A, SD − Trp/X‐α‐Gal/aureobasidin A. (C) The OE‐CsMYB82 and pTRV: CsMYB82 constructs. (D) Quantitative analysis of CsMYB82 overexpression lines (L1, L2, L3, L4, L5, L6, L7, L8) and wild type (WT). The RT‐qPCR data were presented as means ± SD values with three biological replicates. Asterisks indicate statistical significance (**p < 0.01). (E) Petiole injection. The second leaf position was selected for the experiment. (F, G) Confirmation of Virus‐induced gene silencing (VIGS) and OE‐CsMYB82 leaves by RT‐qPCR analysis. #1, #2, #3, #4, #5 and #6 were referred to the distinct pTRV: CsMYB82 leaves in ‘Longjing 43’. WT(Wild‐type) and pTRV2 as controls. OE#1—OE#11 were referred to the distinct OE‐CsMYB82 leaves in ‘Zhongcha 108’. WT(Wild‐type) and empty vector (EV) as controls. The RT‐qPCR data were presented as means ± SD values with three biological replicates. “ns” means no difference and asterisks indicate statistical significance (*p < 0.05, **p < 0.01). (H) Lignin accumulation through phloroglucinol staining in OE‐CsMYB82 leaves. Scale bar = 100 μm. Figure S3: Gene expression analysis in lignin synthesis pathway in pTRV: CsMYB82 and OE‐CsMYB82 leaves. (A) Gene expression analysis in pTRV: CsMYB82 leaves. (B) Gene expression analysis in OE‐CsMYB82 leaves. The RT‐qPCR data were pre [file PBI-24-4725-s003.zip › 3_FigS8.jpg]
